# Supplementary material for: Women’s experience of episiotomy: a qualitative study from China
Source: BMJ Open. 2020 Jul 19;10(7):e033354. doi: 10.1136/bmjopen-2019-033354 (PMC7371141; doi:10.1136/bmjopen-2019-033354)
Supplement: Supplementary data [file bmjopen-2019-033354supp002.pdf]

Characteristics of health care providers

	**Health care providers**
**Age (year)**	
Mean±SD	40.8±5.0
Range	29~52
**Occupation**	
Obstetrician	4
Midwife	5
Community health care provider	3
**Obstetric Institution**	
Municipal MCH Hospital	2
Tertiary general hospital	2
District MCH Hospital	3
Secondary general hospital	3
Community health center	2
**Work experience (year)**	
Mean±SD	16.8±8.2
Range	3~28
