# Supplementary material for: Women’s experience of episiotomy: a qualitative study from China
Source: BMJ Open. 2020 Jul 19;10(7):e033354. doi: 10.1136/bmjopen-2019-033354 (PMC7371141; doi:10.1136/bmjopen-2019-033354)
Supplement: Supplementary data [file bmjopen-2019-033354supp003.pdf]

Themes and illustrative quotes

Themes	Quotes
**Women's views of the procedure vary considerably**	"I barely knew anything about episiotomy before the delivery."(#6, 28 years old, primipara, six months after childbirth)<br>"The doctors didn't inform me about the procedure(episiotomy). After childbirth, the woman in the same delivery ward asked me 'did you get episiotomy' and I reply 'what's the episiotomy?' I didn't know it before and I finally realized what the anesthesia and suturing meant at that time." (#9, 33 years old, primipara, four days after childbirth)<br>"I used to wonder what episiotomy is, and only came to know exactly what it is after childbirth··· at that time [When I was cut] I know it -- Oh, this is episiotomy!" (#14, 28 years old, primipara, one week after childbirth)<br>"At that time, I thought, 'Oh my god! They will certainly cut my vulva. The vulva would be ugly and [its function would be] affected!' It sounds scary." (#28, 21 years old, primipara, six months after childbirth)<br>"I think it is necessary to do episiotomy when it can accelerate the progress of labor. But if the baby can be delivered smoothly, episiotomy should be avoided. After all, it is still a surgery." (#11, 30 years old, primipara, two months after childbirth)<br>"The hospital takes episiotomy as a routine practice during normal vaginal birth. I think if episiotomy can relieve your suffering, routine episiotomy should be recommended. I felt that my perineum recovered soon after episiotomy. On the other hand, episiotomy won't cause any big problems, as long as you move carefully and clean yourself frequently." (#26, 28 years old, primipara, two weeks after childbirth)<br>"The doctor said that my uterine contractions were too weak, but I didn't feel that way. I just needed some time. I don't like the episiotomy at all. I searched episiotomy on the Internet and found its rate in China is excessively high. Many situations are not necessary. The doctors might be afraid of potential risks. I think if there is nothing wrong with the puerpera, the episiotomy should be avoided as far as possible. Previous generation like my mother didn't use episiotomy but they recovered quite well." (#8, 34 years old, primipara, two months after childbirth)
**The pain interferes with daily life for**	"I still feel pain of my perineal wound now and I can feel the difference between the two sides of perineum… the right side with the episiotomy lack skin elasticity… " (#1, 35 years old, multipara, EP with forceps, six

**weeks**	months after childbirth)<br><br>"The wound hurt in the first few days. Five days after delivery, I started to feel better, but I can still feel the pulling or tugging pain at the incision… it was a bit tight." (#26, 28 years old, primipara, two weeks after childbirth)<br><br>" When I tried to sit down, it seems like that I was sitting on a knife. The wound seemed to split. It hurt that much." (#16, 32 years old, primipara, two years after childbirth)<br><br>"The wound split at the six day after birth, then I suffered a lot because it recovered slowly. The pain had continued for half a month and the stiches cannot be absorbed… even now, I am still feeling painful when I am sitting" (#8, 34 years old, two months after childbirth)<br><br>"I had to move slowly because I felt painful when I moved suddenly." （#12, 40 years old, multipara, two months after childbirth）<br><br>"The doctor said I must sleep on the side. It was uncomfortable to sleep one-sided for a long time." (#6, 28 years old, primipara, six months after delivery)<br><br>"At that time (half a month after childbirth), I couldn't sit or squat [because of the horrible pain], and I had to move very slowly." (#8, 34 years old, two months after childbirth)<br><br>"The healing was not very good [of my perineum]… in the first few days, I was fed by my mother. I couldn't sit [because of pain], and I just lay down there. I ate on the bed in the first month. " (#20, 30 years old, primipara, EP with forceps delivery, two months after childbirth)<br><br>"I can't sit because of the pain… in the first month after childbirth, I used the breast pump in a standing position." (#20, 30 years old, primipara, EP with forceps delivery, two months after childbirth)<br><br>"I just felt the wound painful during breastfeeding. Sometimes I felt that my baby couldn't get the nipples, so I just leaned forward a bit, and suddenly, the episiotomy wound began to hurt. After all, there were two cuts in my perineum [The women also got episiotomy at her last childbirth]… and I don't like to lie down [to feed the baby]." (#4, 34 years old, multipara, two months after childbirth)<br><br>"It was very tiring and painful to sit down… I felt my wound was also swollen, and I had to sit on one-side, lean my body to the side without episiotomy. I sat in this way for the breastfeeding within the whole first month… this made my back hurt and sometimes it was really awful." (#28, 21 years old, primipara, six months after childbirth)<br><br>"My wound hurt very much in the first week, and I couldn't peep or poop at all because I couldn't sit on the toilet. Every time using the toilet was

He S, *et al. BMJ Open* 2020; 10:e033354. doi: 10.1136/bmjopen-2019-033354

	like a torture to me. I think that most women who have received an episiotomy would probably have the same problem as me." (#1, 35 years old, multipara, EP with forceps, six months after childbirth)<br><br>"If the wound gets infected because of improperly sterilization during the procedure, it would be very troublesome. The healing will take one to three weeks. In this kind of case, women with episiotomy would be more tortured than those with C-section." (Obstetrician, 28 years of relevant work experience, district MCH hospital.)<br><br>"Catgut (a kind of stitches) absorption varies from people to people, and some women may be allergic··· have catgut rejection. If the sutures cannot be absorbed, it is always a foreign matter in perineum, which will hurt. " (Midwife, 25 years of relevant work experience, district MCH hospital)<br><br>"Episiotomy does have impacts on daily activities, such as breastfeeding. Some women are unwilling to breastfeed while lying down, or they just don't know how to breastfeed while lying down. Sometimes, people would feel anxious because of the pain. The milk secretion could also be affected by the pain." (Midwife, 20 years of relevant work experience, secondary general hospital)<br><br>"Walking and breastfeeding could be affected [by EP]. Episiotomy has a major impact on breastfeeding, because some women have short nipples, and it is difficult for the baby to suck the nipples when in lying position. Thus, it is easier to breastfeed with sitting position (Community health care provider, 17 years of relevant work experience, community health center)
**Long term anxiety is a consequence for some, described as a "psychological shadow"**	"Because of the terrible perineal pain, I asked my husband to resume sexual life a year later. I didn't dare to do it, because I worried the wound would pain again." (#16, 32 years old, primipara, two years after childbirth)<br><br>"We returned to sexual life quite late, about half a year after delivery. The first three or four times [of sexual life] turned out painful because of the wound and vaginal dryness. When my baby was 10 months old, it wasn't painful anymore." (#23, 29 years old, primipara, 14 months after childbirth)<br><br>"Psychologically, I feel that the vagina cannot recover to original state···you feel the vagina is looser than before. And your spouse also has some psychological barriers to postpartum sexual life. I feel that many mothers who undergo episiotomy will have the shadows of sexual life more or less. The psychological shadow might disappear over time, but I

	don't know yet."(#1, 35 years old, multipara, six months after childbirth)<br><br>"The biggest problem for me now is the next delivery. Will I receive an episiotomy again? Will my second vaginal delivery encounter difficulties because of my rough perineum [the wound of episiotomy this time]?" (#3, 32 years old, primipara, EP with forceps, 14 months after childbirth)<br><br>" I don't dare to deliver my second child through normal birth (vaginal delivery). The experience of recovering from the episiotomy was indeed miserable. It really scared me. Maybe not having a second child is better… or maybe I would choose C-section even though it has some negative effects… if I had a vaginal birth again, and an episiotomy again. I cannot imagine what will happen, my vagina would be totally 'useless' for sexual life." (#13, 39 years old, primipara, two years after childbirth)<br><br>"The doctor directly did the episiotomy at my first childbirth. So I gained some childbirth experience and I was always afraid that I would suffer episiotomy again during this childbirth. There was a psychological shadow when I thought of the childbirth… I was worried about these problems such as deliver again, episiotomy again, miserable recovery of episiotomy. Finally, I still got episiotomy again!" (#29, 30 years old, multipara, six months after childbirth)<br><br>"We used the method of withdraw when having sex, because the inside of my perineum was uneven and rough, which hooked the condom and affected intercourse." (#13, 39 years old, primipara, seven years after childbirth)<br><br>"Some women can't have intercourse at all. Once I met a case that the vagina of the women so tight that I can' even put one finger in, due to the scar contracture." (Obstetrician,28 years of relevant work experience, district MCH hospital)<br><br>"What are the impacts of episiotomy on further childbirth? It is true that it may cast a psychological shadow on those women. If the episiotomy wound from first childbirth is infected or she has a severe tear, she won't dare to have another child, or she might choose C-section. Next time she might say: 'I don't want another baby' and 'I want to choose C-section instead of vaginal delivery."(Obstetrician, 28 years of relevant work experience, district MCH hospital)<br><br>"It doesn't matter much because interval between births is generally long. It takes at least one year, right? The skin would recover within a year… it's just that the wound will look ugly but the birth process won't be affected." (Midwife, 25 years of relevant work experience, district MCH hospital)<br><br>"Some people are scar physique (a kind of people who easily have enormous scar). This kind of scar is hard and protuberant so that we fear

	the wound would tear again during the second childbirth. If the scar tears and is sewed up again, it can't heal very well. (Midwife, 25 years of work experience, secondary general hospital)
**Societal norms assume women will not complain**	"I would endure the pain and not mention it. It didn't hurt that much. I could still bear with it... It's normal thing, also the fate of every woman." (#29, 30 years old, multipara, six months after childbirth)<br><br>"Whenever I said I felt sore of the perineal wound, they would say, 'why you still feel painful after 4 months?' It sounds like I shouldn't be sore. Every time my husband said these words, I would response to him, 'you should get a cut and experience the healing process." (#28, 21 years old, primipara, six months after childbirth)<br><br>"I wondered if all the women would have the perineal pain after the childbirth... they [family members], such as my sister in law said that I was a bit low-tolerant... they all had birth experience but they never heard that a puerpera unable to sit down after childbirth... I didn't see a doctor because my families said every woman would experience pain after childbirth, and the doctor also said my wound healed well... At that time, I felt it was so hard to be a woman." (#16, 32 years old, primipara, two years after childbirth)<br><br>"I'm not very low-tolerant... some women are too spoiled to bear any pain and they always groan, which I thought it is meaningless. Nobody could replace your sufferings. It's normal thing, also the fate of every woman." (#29, 30 years old, multipara, six months after childbirth)<br><br>"The suturing process was more painful. I cannot keep unmoved because the anesthetic effects tailed off later. And the doctor kept telling me not to move, saying that he couldn't sew up well if I still move. But it was painful and he was sewing up for a long time because my wound was very big...I couldn't stay still, and I didn't know whether the stitches were done properly. I don't know if it related to my unabsorbed suturing knot, maybe it resulted from my own body condition (some immune factor). " (#20, 30 years old, primipara, EP with forceps delivery, two months after delivery)<br><br>"It was painful during suturing. It was the sharp tingling when I got the cut but the pain of the childbirth was more painful than this. After local anesthetic, it was still very painful and he sewed a few stitches. But I was immersed in the joy of having a baby at that time so I felt the pain could be tolerated···but I was trembling because of pain and the doctor comforted me 'it would be finished soon, you can bear it, yes?" (#23, 29 years old, primipara, 14 months after delivery)<br><br>"I felt painful so much! I thought I really needed some treatments to relive

	the pain but the doctor thought I could endure this kind of pain… I really can't endure it since my wound is very large. I hadn't fallen asleep for several days after childbirth. The pain was so awful!" (#20, 30 years old, primipara, EP with forceps, two months after childbirth)

He S, *et al. BMJ Open* 2020; 10:e033354. doi: 10.1136/bmjopen-2019-033354
