# Supplementary material for: Women’s experience of episiotomy: a qualitative study from China
Source: BMJ Open. 2020 Jul 19;10(7):e033354. doi: 10.1136/bmjopen-2019-033354 (PMC7371141; doi:10.1136/bmjopen-2019-033354)
Supplement: Author's manuscript [file bmjopen-2019-033354.draft_revisions.pdf]

BMJ Open

BMJ Open is committed to open peer review. As part of this commitment we make the peer review history of every article we publish publicly available.

When an article is published we post the peer reviewers' comments and the authors' responses online. We also post the versions of the paper that were used during peer review. These are the versions that the peer review comments apply to.

The versions of the paper that follow are the versions that were submitted during the peer review process. They are not the versions of record or the final published versions. They should not be cited or distributed as the published version of this manuscript.

BMJ Open is an open access journal and the full, final, typeset and author-corrected version of record of the manuscript is available on our site with no access controls, subscription charges or pay-per-view fees (http://bmjopen.bmj.com).

If you have any questions on BMJ Open's open peer review process please email info.bmjopen@bmj.com

BMJ Open

Women's experience of episiotomy: a qualitative study from China

Journal:	*BMJ Open*
Manuscript ID	bmjopen-2019-033354
Article Type:	Research
Date Submitted by the Author:	31-Jul-2019
Complete List of Authors:	He, Siyuan; Fudan University, Maternal, Child and Adolescent Health, School of Public Health; Shanghai Center for Health Promotion,<br>Jiang, Hong; Fudan University, Maternal, Child and Adolescent Health, School of Public Health<br>Qian, Xu; Fudan University, Maternal, Child and Adolescent Health, School of Public Health<br>Garner, Paul; Liverpool School of Tropical Medicine, Centre for Evidence Synthesis in Global Health, Department of Clinical Sciences
Keywords:	QUALITATIVE RESEARCH, PRIMARY CARE, Community gynaecology < GYNAECOLOGY, GYNAECOLOGY

SCHOLARONE™
Manuscripts

I, the Submitting Author has the right to grant and does grant on behalf of all authors of the Work (as defined in the below author licence), an exclusive licence and/or a non-exclusive licence for contributions from authors who are: i) UK Crown employees; ii) where BMJ has agreed a CC-BY licence shall apply, and/or iii) in accordance with the terms applicable for US Federal Government officers or employees acting as part of their official duties; on a worldwide, perpetual, irrevocable, royalty-free basis to BMJ Publishing Group Ltd ("BMJ") its licensees and where the relevant Journal is co-owned by BMJ to the co-owners of the Journal, to publish the Work in this journal and any other BMJ products and to exploit all rights, as set out in our licence.

The Submitting Author accepts and understands that any supply made under these terms is made by BMJ to the Submitting Author unless you are acting as an employee on behalf of your employer or a postgraduate student of an affiliated institution which is paying any applicable article publishing charge ("APC") for Open Access articles. Where the Submitting Author wishes to make the Work available on an Open Access basis (and intends to pay the relevant APC), the terms of reuse of such Open Access shall be governed by a Creative Commons licence – details of these licences and which Creative Commons licence will apply to this Work are set out in our licence referred to above.

Other than as permitted in any relevant BMJ Author's Self Archiving Policies, I confirm this Work has not been accepted for publication elsewhere, is not being considered for publication elsewhere and does not duplicate material already published. I confirm all authors consent to publication of this Work and authorise the granting of this licence.

For peer review only

Title page

Title: Women's experience of episiotomy: a qualitative study from China

***Corresponding author:** Xu Qian, Mailbox 175, 138 Yixueyuan Road, Shanghai, 200032, China; email: xqian@shmu.edu.cn; Tel: +86 021 5427267

All co-authors:

#Siyuan He

Department of Maternal, Child and Adolescent Health, School of Public Health, Fudan University, Shanghai, China.

Shanghai Center for Health Promotion, Shanghai, China.

#Hong Jiang

Department of Maternal, Child and Adolescent Health, School of Public Health, Fudan University, Shanghai, China.

*Xu Qian

Department of Maternal, Child and Adolescent Health, School of Public Health, Fudan

For peer review only

For peer review only - http://bmjopen.bmj.com/site/about/guidelines.xhtml

University, Shanghai, China.

Paul Garner

Centre for Evidence Synthesis in Global Health, Department of Clinical Sciences,

Liverpool School of Tropical Medicine, Liverpool, UK.

#These authors contributed equally to this work.

Word count：3976 words

For peer review only

For peer review only - http://bmjopen.bmj.com/site/about/guidelines.xhtml

ABSTRACT

Objectives To evaluate women's experience, reflections and perspectives of episiotomy.

Design Semi-structured, in-depth individual interviews were used and transcriptions were analyzed using a thematic analysis in Chinese, which we then debated in English to finalize interpretation.

Settings Two community health centers and hospitals in Shanghai.

Participants Purposeful sampling of 30 postpartum women, who had experienced episiotomy, complemented with interviews with providers.

Results Most were primiparous (25/30), and four had forceps. We identified four themes: a) comprehension of the procedure varied considerably; b) The pain and can interfere with daily life for weeks; c) Long term physical and psychological effects can cause anxiety; d) social norms assume women will not complain of pain and should suffer alone.

Conclusion Women experience a wide range of effects from episiotomy. Women receive little or no information in advance and they are concerned about the impact on them as women. These factors, combined with cultural expectations for pain tolerance, make their long-term experience of the procedure worse.

Key words: episiotomy, perineal trauma, women's experience

Strength and Limitations of this study

For peer review only - http://bmjopen.bmj.com/site/about/guidelines.xhtml

- This study was one of the very few qualitative studies to understand details of women's experience, reflections and perspectives of episiotomy after childbirth
- To enrich the information, we interviewed women of different times after episiotomy and included health care providers' responses as supplement.
- Some women were interviewed more than six months after childbirth, which might introduce the recall bias.
- We interviewed the women in different times after childbirth instead of follow up every woman from delivery to recovery.

For peer review only

For peer review only - http://bmjopen.bmj.com/site/about/guidelines.xhtml

INTRODUCTION

Episiotomy aims to reduce the risk of lacerations during deliveries, shorten delivery and prevent damage to the pelvic floor; but it can cause bleeding, infection, and long-term dyspareunia[1] Over the last 15 years, the global shift has been towards restrictive use. Many international institutions and professional societies such as World Health Organization and American College of Obstetricians and Gynecologists recommend to use episiotomy only when there is the strict clinical indication, instead of the routine use policy[2-5]. And practice in most European countries[6] shows rates have fallen. However, episiotomy rates are still reported high in some countries (If not specified, the rates of episiotomy refer to women with vaginal childbirth), for example 53.2% in Chile[7] (multi-centers data), 73% in Lebanon [8] (teaching hospital data), and 92% in Cambodia[9] (single hospital study).

China still has a high episiotomy rate. In the last decade, hospital data reported levels of 47.4% to 84.7% [10-13]; and, from multi-centers studies, from 41.2% to 69.7%[14, 15].Although the Chinese national obstetric guideline now recommends restrictive use of episiotomy in 2016[16], implementation has yet to take effect. With more than 15 million births every year, there could be as many as 6 million episiotomies a year, given a Caesarian Section rate of 39.0%.

There is very few research that has examined women's perceived experience worldwide, and trials inadequately consider women's preferences, views on the procedures, or the outcomes that are important to them[17].We therefore carried out this qualitative study to

For peer review only - http://bmjopen.bmj.com/site/about/guidelines.xhtml

explore women's experience, reflections and perspectives regarding episiotomy from a mixed population who resident in Shanghai. The population of Shanghai is 24.2 million (2016), with a policy of routine episiotomy from 1999; and rates of around 35.8%~82.22%, data from 2012 to 2014[18-20]

METHODS

Approach, setting and sampling strategy

Semi-structured, in-depth individual interviews were used and transcriptions were analyzed using a thematic analysis in this study. The details of the methods were reported according to the SRQR reporting checklist (see Supplementary File 1)[21]. We conducted the study in two community health centers in Shanghai. Community health center provides pregnancy registration and postnatal (home visit) and child health care for the clients in its service catchment area, while clinical services for childbirth are provided by higher level hospitals according women's preferences and health needs. The two community health centers we used are where we have worked before and staff are familiar with us. One in Pudong District, east of Shanghai (1459 pregnant women registered in 2017); and the other in Xuhui District, west of Shanghai (775 pregnant women registered in 2017). Purposeful sampling strategy was used in our study. We sought women who were over 18 years old and had experienced episiotomy in their last birth. We recruited women from three different postpartum periods (within two weeks, no

For peer review only

For peer review only - http://bmjopen.bmj.com/site/about/guidelines.xhtml

more than six months and six months above after childbirth) and also considered about their ages, parity, and level of hospitals who had childbirth.

We also interviewed doctors, midwives and community health care providers with over three years work experience in maternal health from different level of service delivery points. Individual interviews with postpartum women were stopped when data saturation was reached. The primary researcher (SH) carried out the interviews, under the guidance of the supervisors (HJ and XQ). SH is a master student, who had received training of qualitative study and had a six-month work placement with the MCH administration.

Ethical approval

All participants were informed about the research purpose and contents. Interviews were conducted after written informed consent obtained from each participant. The research obtained the approval from the Institutional Review Board in School of Public Health, Fudan University.

Patient and public involvement

We invited some women to give suggestions at the stage of study design, as a way of patient and public involvement (PPI), in order to ensure this study was women focused. We collected women's comments on the public internet forum and interviewed some women before we designed the interview guide. According to women's opinions, we also made several revisions to the interview guide. In order to increase public attention on this "embarrassing and mysterious" topic, we will write an editorial or a blog in the future.

For peer review only - http://bmjopen.bmj.com/site/about/guidelines.xhtml

Further studies could also involve women in data analysis such as check the accuracy of the results and interpretations. We didn't try to involve women in the recruitment and conduct of the study because we thought the negative comments might be excessively enlarged.

Data collection

We approached women by accompanying health staff during postpartum home visits or when women brought their children for child health checkup in community health centers between September 2017 and March 2018. We used an interview guide based on the literatures and our research group discussions (Table 1)[22-24]; we piloted the form with four postpartum women till little change was needed . The piloted data were also included in our analysis as it was consistent with the main sample. Interviews were conducted in private rooms in the community health centers, hospitals and interviewees' homes and all women provided signed consent. Interviews were in Chinese and recorded with permission. For the health providers, they were recommended by relevant administrators and invited to this study. They were interviewed at a private room in their workplace.

For peer review only - http://bmjopen.bmj.com/site/about/guidelines.xhtml

Table 1 Interview guide

Could you tell me your experience after episiotomy? (Probe: discomfort, pain, swelling)
Did episiotomy impact on your daily life? (Probe: walking, sitting, breastfeeding, baby care, sexual life, medication, mood)
How did you deal with your sufferings or problems? (Probe: medical services usage)
Are there some long-lasting effects of episiotomy you have noticed?

Data analysis

Medical master students transcribed interviews, and interviewers checked them for accuracy. We used NVivo8.0 (QSR) software for thematic analysis [25], with two researchers (SH and YC) reading all the transcripts and coding concepts or themes. Initial themes and quotes were translated into English and checked by XQ. When we found a theme, where was no direct translation to English, the team discussed the words carefully in Chinese and English to gain a common understanding of meaning and cultural context. The research team consisted of three bilingual speakers (SH, HJ, XQ) and one native English speaker (PG). After discussion, an initial thematic framework was set up and SH coded all the transcripts. The themes, descriptions and corresponding quotes were checked by HJ and PG. To avoid cluttering the text with quotes, the main illustrative quotes are included in appendices.

For peer review only

For peer review only - http://bmjopen.bmj.com/site/about/guidelines.xhtml

The health professional's responses were grouped against the emergent themes from the women's interviews and included as a separate section within a theme.

Reflexivity

The research team included people that had performed, repaired and experienced episiotomy (HJ, QX, PG). Evaluating episiotomy is a policy interest for all authors, and three have authored the Cochrane review examining this topic (HJ, QX, PG). The Cochrane review included comments that consumer views on the procedure are important for policy.

RESULTS

We interviewed 30 postpartum women (Table 2) from the four types of hospitals, with all different episiotomy practices. In addition, we interviewed twelve health staff including obstetricians (4), midwives (6), and community health-care providers who provide home visit services (2). The doctors and midwives came from a range of hospitals with practices from routine to restrictive episiotomy: Municipal MCH Hospital (2), Tertiary general hospital (2), District MCH Hospital (3) Secondary general hospital (3). Four themes emerged and the selected quotes of each theme were shown at the end of the results (Table 3). The complete illustrative quotes were shown in the Supplementary File 2.

For peer review only - http://bmjopen.bmj.com/site/about/guidelines.xhtml

Table 2 Characteristics of the Postpartum Women

	Women
**Age**	
Mean	30.1±3.8
Range	21~40
**Parity**	
Primipara	25
Multipara	5
**Mode of delivery**	
Episiotomy	26
Episiotomy with forceps	4
**Interview time**	
Within two weeks after childbirth	7
No more than six months after childbirth	9
Six months above after childbirth	14

For peer review only

Comprehension of the procedure varied considerably

Some women knew little about the procedure. Some women's understanding was shaped by their experience in real time and the people around them. For example, one woman even heard about it from the woman being wheeled out of the delivery ward;

For peer review only - http://bmjopen.bmj.com/site/about/guidelines.xhtml

another woman had heard the word, but was not clear what it meant, until it happened: “The doctor cut me and then I thought, Yes, now I know what it is!” Besides these uninformed women, some women simply knew that “episiotomy is a cut to the vagina” and stated it felt frightening but knew little more.

Other women seemed to be more informed, from a variety of sources: online resources, discussion with other women, and some from doctors. Some of these more informed women reflected on the clinical equipoise in their conversations: fear of the procedure and the damage to the vagina but accepting that the procedure would accelerate the progress of labor, guarantee the safety of the baby, and avoid tears.

Their individual experience seemed important in shaping their views: one woman accepted routine episiotomy was required, and another multipara who had an episiotomy with her first childbirth requested it for her second delivery. Women with few problems seemed more accepting of the need for the procedure. However, those who had a miserable experience seemed more likely to complain the negative effects and question the need for an episiotomy.

The pain and discomfort can interfere with daily life for weeks

Women reported varying degrees of pain and discomfort from episiotomy, and sometimes this lasted for months. Most women felt painful for two weeks and they only described the pain as "a little pain or discomfort ", but a few women felt pain months after childbirth, three reporting this as “intolerable” for more than one month. These women

For peer review only - http://bmjopen.bmj.com/site/about/guidelines.xhtml

with terrible pain also mentioned they had some problems of suturing including tight suturing, irritation from the stitches or the wound gaping, and two of them felt much better as soon as the stitches removed.

Fear of pain and split of the episiotomy wound did restrict movement in most women. Some women stated they were conscious of the wound and avoiding pain, so had to walk or move slowly, or avoid contact as the wound hurt when pressed. Some had to sit or lie on one-side or stay in one position for a long time to avoid pain, and this made them tired and uncomfortable. Three women with poor healing condition complained that they could not sit down for a minute because of the horrible pain, which made them incapable of looking after their babies and themselves.

For those with pain, several noted this interfered breastfeeding, and most women believed the wound of episiotomy affected breastfeeding somehow. Since women liked to feed baby whilst sitting, if this was painful then they struggled to feed. Some of them learned to breastfeed by lying down or using breast pump in a standing position. Other women sat painfully and arduously to feed their babies, which increased the difficulty and fatigue of breastfeeding.

The pain interfered with defecation, with increasing pain and the sensation the wound was about to split whilst defecating. Just sitting or squatting was also hard. This fear of pain or split led to the unwilling of defecation, in women already with constipation or hemorrhoids, making matters worse. Furthermore, two women even mistakenly regarded the pain of episiotomy as the pain of hemorrhoids for weeks.

For peer review only - http://bmjopen.bmj.com/site/about/guidelines.xhtml

The interviews with the health providers gave a slightly different pattern. They considered that perineal pain from episiotomy is usually tolerable and does not last long, unless there is something wrong such as infection or unabsorbed suturing. The doctors did not mention the effects with breastfeeding. On the other hand, the community health care providers and midwives confirmed the difficulties of breastfeeding but commented that some women had to breastfeed in a painful sitting position because they didn't know how to feed baby in any other way; and that the sitting posture was the proper way for the baby to suck mother's nipples. As for postpartum constipation and pain of defecation, health professionals were aware of these problems.

Long term physical and psychological effects can cause anxiety

Several women used the word "psychological shadow" (心理阴影) cast by the long-term effects from episiotomy. The Chinese word implies a negative experience of suffering or torment that leads to a dread or worries of the future-a bit like the experience of war or a tumultuous personal event. In these women, the conversations were extensive, the women were clearly troubled about what would happen with sex or if they were to have another baby.

The concern about sex was apparent in those women who resumed sex: they mentioned the uneven or rough skin of perineal wound and painful intercourse, which affected the enjoyment of sexual life. One woman said the pain in the sexual life might came from her psychological factors and her anxiety. Another woman asked her husband to await till one year after childbirth, because she suffered severe pain of episiotomy for nearly two

For peer review only - http://bmjopen.bmj.com/site/about/guidelines.xhtml

months and feared sexual life might make her back to the nightmare again. Some responses around resumption of sex and the "psychological shadow" included beliefs that their vagina was damaged and loose and may not ever recover. For these women, they were unwilling to start sex and again, and were always conscious that there had been damage done to their vagina.

The "psychological shadow" also impacted on how some women viewed a possible subsequent pregnancy. Some expressed concern as to whether the episiotomy wound would hinder the process of next vaginal delivery; whether the wound will split again in the next vaginal delivery; whether they will get episiotomy again. This influenced how they viewed subsequent pregnancies and how they might deliver: at least one woman reported that next time she would ask for a cesarean section to avoid episiotomy. One woman said, "if I had a vaginal birth again, and an episiotomy again. I cannot imagine what will happen, my vagina would be totally 'useless' (没用) for sexual life." The interviews indicated a high degree of anxiety about the long-term physical consequences, and reflect how this then itself causes further anxiety.

The health providers were aware of physical abnormalities following episiotomy and psychological concerns about sexual life; most dismissed concerns about subsequent deliveries: one midwife thought the hard scar is easy to tear again if episiotomy isn't done in advance. However, most of them conceded that miserable recovery experience of episiotomy can cause women's fear and anxiety of next delivery.

Social norms assume women will not complain of pain and should suffer alone

For peer review only - http://bmjopen.bmj.com/site/about/guidelines.xhtml

Women indicated that their family members and health professionals regarded pain and discomfort as a normal part of childbirth and the puerperal period. This pain and discomfort are expected and will gradually disappear without treatment. Whilst women accepted this, it appeared that this expectation did not take into account the more substantive pain, discomfort and interference with daily life associated with episiotomy (see theme 1) and this is distressing for some women. Some women felt the expectations that they should not complain much about the "slight and temporary discomforts" but to be strong and endure the pain or discomfort by themselves.

The family members of a few women expected them to endure the "non-severe discomfort". Indeed, a few women were frightened that if they complained too much they would be judged as "being low-tolerant"(娇气). This word is a pejorative personality trait, which means a person exaggerate something that is slightly uncomfortable. This word refers to people who have "weak minds" and who are rather cowardly. When some women with normal vaginal birth expressed or complained the postpartum suffering too much, their families thought their discomforts were slight, that the women should have endured this, and were at risk of this weak character trait of being "low-tolerant". One woman said her family thought it was too exaggerated that a woman with normal birth cannot sit for a minute: they had experienced normal birth and didn't encounter anything terrible. When this judgement happened, the women felt upset and unwilling to speak out their needs, suffering alone. Surprisingly, another interviewed woman even regarded the tolerance of pain as women's own destiny. Thus, the social norms make some women

For peer review only - http://bmjopen.bmj.com/site/about/guidelines.xhtml

"suffer alone" and stops them from seeking care.

Indeed, these social norms about tolerating pain also manifest in the way health care was provided. Most women thought suturing is more painful than being cut; yet some doctors did not check whether women were always effectively anaesthetized during the suturing. One woman complained of pain during suturing but was told to "wait-it will be finished soon"; and another was told to stay still. One woman reported the pain was so severe she did move when being sutured, and then blamed herself for the subsequent healing problems because she had moved. The expected tolerance of pain extended to pain relief: a woman asked for pain relief after childbirth but be refused by the doctors with the reason "the level of the pain can be tolerated".

Table 3 Summary of themes with selected quotes

Themes	Selected quotes
Comprehension of the procedure varied considerably	"The doctors didn't inform me about the procedure(episiotomy). After childbirth, the woman in the same delivery ward asked me 'did you get episiotomy' and I reply 'what's the episiotomy?' I didn't know it before and I finally realized what the anesthesia and suturing meant at that time." (#9, 33 years old, primipara, four days after childbirth)<br><br>"I think it is necessary to do episiotomy when it can accelerate the progress of labor. But if the baby can be delivered smoothly, episiotomy should be avoided. After all, it is still a surgery." (#11, 30 years old, primipara, two months after

For peer review only - http://bmjopen.bmj.com/site/about/guidelines.xhtml

	childbirth)<br><br>“The doctor said that my uterine contractions were too weak, but I didn't feel that way. I just needed some time. I don't like the episiotomy at all. I searched episiotomy on the Internet and found its rate in China is excessively high. Many situations are not necessary. The doctors might be afraid of potential risks. I think if there is nothing wrong with the puerpera, the episiotomy should be avoided as far as possible. Previous generation like my mother didn’t use episiotomy but they recovered quite well." (#8, 34 years old, primipara, two months after childbirth)
**The pain and discomfort can interfere with daily life for weeks**	"The healing was not very good [of my perineum], because the incision was relatively big. I went to emergency room twice within the first month [after childbirth]. The reason was the wound got infected. After removing the stiches, I was getting recovered from the cut. In the first few days, I was fed by my mother. I couldn't sit, and I just lay down there. I ate on the bed in the first month. " (#20, 30 years old, primipara, EP with forceps delivery, two months after childbirth)<br><br>"It was very tiring and painful to sit down… I felt my wound was also swollen, and I had to sit on one-side, lean my body to the side without episiotomy. I sat in this way for the breastfeeding within the whole first month …. This made my back hurt and sometimes it was really awful." (#28, 21 years old, primipara, six

For peer review only - http://bmjopen.bmj.com/site/about/guidelines.xhtml

	months after childbirth)<br><br>"…. If the wound gets infected because of improperly sterilization during the procedure, it would be very troublesome. The healing will take one to three weeks. In this kind of case, women with episiotomy would be more tortured than those with C-section." (Obstetrician, 28 years of relevant work experience, district MCH hospital.)<br><br>"Episiotomy does have impacts on daily activities, such as breastfeeding. Some women are unwilling to breastfeed while lying down, or they just don't know how to breastfeed while lying down. Sometimes, people would feel anxious because of the pain. The milk secretion could also be affected by the pain." (Midwife, 20 years of relevant work experience，secondary general hospital)
**Long term physical and psychological effects can cause anxiety**	"We used the method of withdraw when having sex, because the inside of my perineum was uneven and rough, which hooked the condom and affected intercourse." (#13, 39 years old, primipara, seven years after childbirth)<br><br>"Because of the terribly perineal pain, I asked my husband to resume sexual life a year later. I didn't dare to do it, because I worried the wound would pain again." (#16, 32 years old, primipara, two years after childbirth)<br><br>" I don't dare to deliver my second child through normal birth (vaginal delivery). The experience of recovering from the episiotomy was indeed miserable. It really scared me. Maybe not having a second child is better… or maybe I would

For peer review only - http://bmjopen.bmj.com/site/about/guidelines.xhtml

	choose C-section even though it has some negative effects. …… if I had a vaginal birth again, and an episiotomy again. I cannot imagine what will happen, my vagina would be totally 'useless' for sexual life." (#13, 39 years old, primipara, two years after childbirth)<br><br>"It doesn't have a lot of affection, because interval between births is generally long. It takes at least one year, right? The skin would recover within a year….it's just that the wound will look ugly but the birth process won't be affected." (Midwife, 25 years of relevant work experience, district MCH hospital)<br><br>"Some people are scar physique (a kind of people who easily have enormous scar). This kind of scar is hard and protuberant so that we fear the wound would tear again during the second childbirth. If the scar tear and was sew up again, it can't heal very well. (Midwife, 25 years of work experience, secondary general hospital)
**Social norms assume women will not complain of pain and should suffer alone**	"I wondered if all the women would have the perineal pain after the childbirth…They [family members], such as my sister in law said that I was a bit low-tolerant, ...they all had birth experience but they never heard that a puerpera unable to sit down after childbirth. At that time, I felt it was so hard to be a woman." (#16, 32 years old, primipara, two years after childbirth)<br><br>"No, I'm not very low-tolerant. Even if it hurts, I would endure the pain and not mention it. It didn't hurt that much. I could still bear with it. Some women are too

For peer review only - http://bmjopen.bmj.com/site/about/guidelines.xhtml

	spoiled to bear any pain and they always groan, which I thought it is meaningless. Nobody could replace your sufferings. It's normal thing, also the destiny of every woman." (#29, 30 years old, multipara, six months after childbirth)<br><br>"It was painful during suturing. It was the sharp tingling when I got the cut but the pain of the childbirth was more painful than this. After local anesthetic, it was still very painful and he sewed a few stitches. But I was immersed in the joy of having a baby at that time so I felt the pain could be tolerated…but I was trembling because of pain and the doctor comforted me 'it would be finished soon, you can bear it, yes?" (#23, 29 years old, primipara, 14 months after delivery)<br><br>"I felt painful so much! I thought I really needed some treatments to relive the pain but the doctor thought I could endure this kind of pain……I really can't endure it since my wound is very large. I hadn't fallen asleep for several days after childbirth. The pain was so awful!" (#20, 30 years old, primipara, EP with forceps, two months after childbirth)

For peer review only

DISCUSSION

Our study described women's sufferings, reflections and perspectives regarding episiotomy after childbirth. Women were inadequately informed about episiotomy,

For peer review only - http://bmjopen.bmj.com/site/about/guidelines.xhtml

especially for its consequences. However, women did suffer the consequences of episiotomy including pain, interfered daily life, long-lasting anxiety and even unfair social norms. Women's views of episiotomy were not only shaped by their knowledge's bout episiotomy, but also the whole lived experience including the delivery and postpartum recovery.

Strengths and Limitations

There are very few qualitative studies of this kind, and the ones we have identified do not differentiate between episiotomy alone and perineal trauma (including episiotomy and severe tear). Other studies are usually in hospital setting and concerned with shorter term consequences of episiotomy[26-29]. The limitation of our study included, first, some women were interviewed more than six months after childbirth, which might introduce the recall bias. Second, we interviewed the women in different times after childbirth instead of follow-up every woman from delivery to recovery, so we cannot know the duration of some women's sufferings and the shift of their understating of episiotomy.

We invited a few women to participate in the study design, which is an attempt of patient involvement. In this way, we can find women's significant opinions about episiotomy that we ignored before, meanwhile, some complaints about episiotomy might be also exaggerated.

Episiotomy brings extensive physical discomforts and life troubles to solve. In this study, we confirmed women did suffer the perineal pain or discomfort, consistent with qualitative

For peer review only - http://bmjopen.bmj.com/site/about/guidelines.xhtml

and quantitative studies[24, 30, 31]. Besides, women in this study complained more about the unabsorbed stitches or split of stitches. Many reviews also indicate there are a part of women need removal or re-suture services due to factors of materials or skills[32, 33]. Women in this study also reported episiotomy limited postpartum daily activities including sitting, breastfeeding, defecation, and intercourse. Whilst these are well recognized in the studies about perineal trauma, we have found less about how this interferes with breastfeeding. Chou mentioned the perineal pain can interfere the initiation of breastfeeding[34] and Persico found the exclusive breastfeeding rate of women with episiotomy in first day after delivery was lower than the women with intact perineum[35]. These physical symptoms or morbidity can also cause psychological burden or anxiety. A study in Jordan reported there was a significant association between post-partum depression and 15 health problems of obstetric, gynecologic (i.e. episiotomy pain, infection), and general health conditions (i.e. fatigue, headache)[36]. These physical problems might have cumulative effects, as a prospective study indicated high burden of breastfeeding problems alone or with co-morbid physical problems was significantly associated with poor maternal mood at 8 weeks. This kind of influence is also obvious of the issues about sexual life and further delivery. Our findings highlighted that some women with episiotomy feared, or even wanted to avoid another pregnancy because of the painful experience in postpartum period. This concerns is also reported in other qualitative studies [37, 38], and the association between postpartum sexual life and episiotomy are researched a lot[39, 40]. Researches to date have not illustrated this

For peer review only - http://bmjopen.bmj.com/site/about/guidelines.xhtml

concern about subsequent births.

In this study, women 's understandings of episiotomy were mainly shaped by their experience and the people around them, because of the inadequate information from health system. Most qualitative studies reported the inadequate informed consent of episiotomy. One study mentioned half of interviewed women didn't receive any information about the procedure before or during childbirth [27] Another study reported that the procedure was easily informed and even practiced directly without any words[28]. Some women even did not know whether an episiotomy or spontaneous tear was done, and only noticed a greater discomfort during suturing[26]. Women particularly lacked the knowledges about the consequences of episiotomy in our study, one qualitative study about perineal trauma also identified the similar theme “being unaware of the episiotomy's consequences”[27].

While interviewed women knew little about the consequences of episiotomy, women's families even some health professionals in this region also showed a poor understanding of the consequences of episiotomy. Both women's families and health staffs over-simplified women's health status. In their opinions, women don't have serious problems and can recover quickly after normal childbirth (including vaginal childbirth with episiotomy). This opinion is consistent with a systematic mixed studies review about perineal trauma reported the theme “normalization and feeling dismissed”, which means women's health problems are regarded as a normal consequence after childbirth and their questions keep unanswered by health professionals[41]. Furthermore, we found

For peer review only - http://bmjopen.bmj.com/site/about/guidelines.xhtml

families made unfair judgments according their own childbirth experience, making women more upset, annoyed and even unlikely to speak out their suffering again. Inappropriate expectation and response from social norms adds more negative emotion and distress on postpartum women. Some studies reported women felt frustrated and abandoned because of the "dismissed by health care providers"[42, 43]. This social norm can hinder women from seeking medical services, and make women had to suffer alone.

The study also raised the interplay between physical injury and pain, the societal expectations that this was normal, and the women's personal anxieties about anticipated genital damage and pain with sex. When a woman with sufferings are not expected to complain, the anxiety this causes then has no release and makes matters worse. These factors and interactions are particularly important in China where the episiotomy rates remain high, and where the country is transforming from one-child to two-child policy.

CONCLUSION

Women were inadequately informed about episiotomy, but did suffer the consequences of episiotomy including pain, interfered daily life, long-lasting effects. These were compounded by social norms that expect them not to complain and longer-term anxiety about the physical and psychological effects on them as women. Further health practice is needed to increase awareness of the consequences of episiotomy and improve the follow-up services; assisting women recover comprehensively from the traumatic

For peer review only - http://bmjopen.bmj.com/site/about/guidelines.xhtml

childbirth.

Acknowledgments The authors thank the valuable contribution of the women and health care providers who participated in this study. The authors are very grateful to Qinjin Huang for her arrangements of study settings. The authors would like to thank Chunyi Gu and Yao Chen for the professional guidance and data analysis. The authors would also like to thank the following health staffs for their assistance of recruitment: Xiaoxing Dai, Jiali Zhang, Hongxia Cui, Linlin Li.

Author Contributions SH, HJ and XQ designed the study and analyzed data. SH and HJ drafted the paper, and XQ revised it. PG commented and edited the article. SH and HJ contributed equally to this study, who should be regarded as co-first authors. All authors have verified and approved the final version of the abstract for publication.

Competing interests None declared.

Funding This work received support from the Effective Health care Research Consortium, funded by UK aid from the UK government for the benefit of developing countries (Grant: 5242)

Details of ethics approval All participants were informed about the research purpose and contents. Interviews were conducted after written informed consent obtained from each participant. The research obtained the approval from the Institutional Review Board in School of Public Health, Fudan University (ID: 2017-12-0648)

For peer review only - http://bmjopen.bmj.com/site/about/guidelines.xhtml

Provenance and peer review Not commissioned; externally peer reviewed.

Data sharing statement No additional data are available.

Open access This is an open access article distributed in accordance with the Creative Commons Attribution Non Commercial (CC BY-NC 4.0) license, which permits others to distribute, remix, adapt, build upon this work non-commercially, and license their derivative works on different terms, provided the original work is properly cited, appropriate credit is given, any changes made indicated, and the use is non-commercial. See: http:// creative commons. org/licenses/by-nc/4.0/.

References:

1. Muhleman MA, Aly I, Walters A, et al. To cut or not to cut, that is the question: A review of the anatomy, the technique, risks, and benefits of an episiotomy. *Clin Anat* 2017;30:362-72.

2. WHO recommendations: intrapartum care for a positive childbirth experience. Geneva: World Health Organization; 2018;150-155.

3. Overview | Intrapartum care for healthy women and babies | Guidance | NICE. Avaulable at: https://www.nice.org.uk/guidance/cg190. (Accessed 13 Jul 2019).

4. ACOG. ACOG Practice Bulletin. Episiotomy. Clinical Management Guidelines for Obstetrician-Gynecologists. Number 71, April 2006. *Obstet Gynecol* 2006;107:957-62.

5. RCOG. The Management of Third- and Fourth-Degree Perineal Tears. Green-top Guideline No. 29.(June. 2015).

6. Blondel B, Alexander S, Bjarnadottir RI, et al. Variations in rates of severe perineal tears and episiotomies in 20 European countries: a study based on routine national data in Euro-Peristat Project. *Acta Obstet Gynecol Scand* 2016;95:746-54.

7. Binfa L, Pantoja L, Ortiz J, et al. Midwifery practice and maternity services: A multisite descriptive study in Latin America and the Caribbean. *Midwifery* 2016;40:218-25.

8. Kaddoura R, DeJong J, Zurayk H, et al. Episiotomy practice in the Middle East: A Lebanese teaching tertiary care center experience. *Women Birth* 2019;32:223-228.

9. Schantz C, Sim KL, Ly EM , et al. Reasons for routine episiotomy: A mixed-methods study in a large maternity hospital in Phnom Penh, Cambodia. *Reprod Health Matters* 2015;23:68-77.

For peer review only - http://bmjopen.bmj.com/site/about/guidelines.xhtml

10. Wang Q, Zheng S, Ni Y, et al. The effect of labor epidural analgesia on maternal－fetal outcomes: a retrospective cohort study. *Arch Gynecol Obstet* 2018;298:89-96.

11. Tung CW, Cheon WC, Tong WM, et al. Incidence and Risk Factors of Obstetric Anal Sphincter Injuries after Various Modes of Vaginal Deliveries in Chinese Women. *Chin Med J (Engl)* 2015;128:2420-5.

12. Yang X, Zhang HX, Yu HY, et al. The prevalence of fecal incontinence and urinary incontinence in primiparous postpartum Chinese women. *Eur J Obstet Gynecol Reprod Biol* 2010;152:214-7.

13. Zhao Y, Xiao M, Tang F, et al. The effect of water immersion delivery on the strength of pelvic floor muscle and pelvic floor disorders during postpartum period. *Medicine (Baltimore)* 2017;96:8124.

14. Zhu L, Li L, Lang J, et al. Prevalence and risk factors for peri- and postpartum urinary incontinence in primiparous women in China: a prospective longitudinal study. *Int Urogynecol J* 2012;23:563-72.

15. Liang L, Min L, Li N. Survey on episiotomy among puerperal women in vaginal delivery from 2011 to 2014. *Maternal and Child Health care of China* 2015;07:1087-9.

16. Obstetrics Group, Obstetrics and Gynecology Society, Chinese Medical Association. Clinical guidelines for operative vaginal delivery (2016). *Chin J Obstet Gynecol* 2016;51:565-7.

17. Jiang H, Qian X, Carroli G, et al. Selective versus routine use of episiotomy for vaginal birth. *Cochrane Database Syst Re* 2017;8:2: CD000081.

18. Jin W, He X, Liu P, et al. Maternal and Neonatal Outcomes in Water Birth. *Journal of Practical Obstetrics and Gynecology* 2009;25: 669-71

19. Ye Q，Niu L，Zhao L. Investigation of Puerpera's Pelvic Floor Function and Early Effect of Obstetric Factors on Pelvic Floor Function. *Journal of Practical Obstetrics and Gynecology* 2016;32:843-6

20. Zhuang W, Li Y, Wu N, et al. Effects of Midwife Hierarchical Management mode on Improving the Quality Control of the Delivery Room. *Nurs J Chin PLA* 2013; 30:52-4.

21. O'Brien BC, Harris IB, Beckman TJ, et al. Standards for reporting qualitative research: a synthesis of recommendations. *Acad Med* 2014, 89:1245-51.

22. Tully KP, Stuebe AM, Verbiest SB. The fourth trimester: a critical transition period with unmet maternal health needs. *Am J Obstet Gynecol* 2017;217:37-41.

23. Fahey JO, Shenassa E. Understanding and Meeting the Needs of Women in the Postpartum Period: The Perinatal Maternal Health Promotion Model. *J Midwifery Womens Health* 2013;58:613-21.

24. Dudley L, Kettle C, Waterfield J, et al. Perineal resuturing versus expectant management following vaginal delivery complicated by a dehisced wound (PREVIEW): a nested qualitative study. *BMJ Open* 2017;7:e13008.

25. Burnard P, Gill P, Stewart K, et al. Analysing and presenting qualitative data. *Br Dent J* 2008;204:429-32.

For peer review only

For peer review only - http://bmjopen.bmj.com/site/about/guidelines.xhtml

26. Wey CY, Salim NR, Junior HPDO, et al. The practice of episiotomy: a qualitative study on perceptions of a group of women. *Online Brazilian Journal of Nursing* 2011;10;2. http://www.objnursing.uff.br/index.php/nursing/rt/printerFriendly/3332/html_2

27. Dengo VAR, Dos Santos Silva R, Souza SRRK, et al. Puerperal women's perceptions about episiotomy. *Cogitare Enferm* 2016;21:1-8.

28. Figueiredo G, Barbieri M, Gabrielloni MC, et al. Episiotomy: perceptions from adolescent puerperae. *Invest Educ Enferm* 2015;33:365-73.

29. Previatti JF, de Souza KV. [Episiotomy: focusing women's view]. *Rev Bras Enferm* 2007;60:197-201.

30. Gün İ, Doğan B, Özdamar Ö. Long- and short-term complications of episiotomy. *Turk J Obstet Gynecol* 2016;13:144-8.

31. Manresa M, Pereda A, Bataller E, et al. Incidence of perineal pain and dyspareunia following spontaneous vaginal birth: a systematic review and meta-analysis. *Int Urogynecol J* 2019;30:853-868.

32. Kettle C, Dowswell T, Ismail KM. Continuous and interrupted suturing techniques for repair of episiotomy or second-degree tears. *Cochrane Database Syst Rev* 2012;11: CD000947.

33. Selo-Ojeme DO, Okonkwo CA, Atuanya C, et al. Single-knot versus multiple-knot technique of perineal repair: a randomised controlled trial. *Arch Gynecol Obstet* 2016;294:945-52.

34. Chou D, Abalos E, Gyte GM, et al. Drugs for perineal pain in the early postpartum period (Protocol). *Cochrane Database Syst Rev* 2016;3: CD007734.

35. Persico G, Vergani P, Cestaro C, et al. Assessment of postpartum perineal pain after vaginal delivery: prevalence, severity and determinants. A prospective observational study. *Minerva Ginecol* 2013;65:669-78.

36. Safadi RR, Abushaikha LA, Ahmad MM. Demographic, maternal, and infant health correlates of post-partum depression in Jordan. *Nurs Health Sci* 2016;18:306-13.

37. İlhan G, Verit Atmaca FF, Eken M, et al. What is Turkish women's opinion about vaginal delivery? *Turk J Obstet Gynecol* 2015;12:75-8.

38. Salmon D. A feminist analysis of women's experiences of perineal trauma in the immediate post-delivery period. *Midwifery* 1999;15:247-56.

39. Fodstad K, Staff AC, Laine K. Sexual activity and dyspareunia the first year postpartum in relation to degree of perineal trauma. *Int Urogynecol J* 2016;27:1513-23.

40. Klein MC, Gauthier RJ, Robbins JM, et al. Relationship of episiotomy to perineal trauma and morbidity, sexual dysfunction, and pelvic floor relaxation. *Am J Obstet Gynecol* 1994;171:591-8.

41. Crookall R, Fowler G, Wood C, et al. A systematic mixed studies review of women's experiences of perineal trauma sustained during childbirth. *J Adv Nurs* 2018 May 23. DOI: 10.1111/jan.13724

42. Priddis H, Schmied V, Dahlen H. Women's experiences following severe perineal trauma: a

For peer review only - http://bmjopen.bmj.com/site/about/guidelines.xhtml

qualitative study. *BMC Womens Health* 2014;14:32.

43. Williams A, Lavender T, Richmond DH, et al. Women's experiences after a third-degree obstetric anal sphincter tear: a qualitative study. *Birth* 2005;32:129-36.

For peer review only

For peer review only - http://bmjopen.bmj.com/site/about/guidelines.xhtml

Reporting checklist for qualitative study.

Based on the SRQR guidelines.

Instructions to authors

Complete this checklist by entering the page numbers from your manuscript where readers will find each of the items listed below.

Your article may not currently address all the items on the checklist. Please modify your text to include the missing information. If you are certain that an item does not apply, please write "n/a" and provide a short explanation.

Upload your completed checklist as an extra file when you submit to a journal.

In your methods section, say that you used the SRQRreporting guidelines, and cite them as:

O'Brien BC, Harris IB, Beckman TJ, Reed DA, Cook DA. Standards for reporting qualitative research: a synthesis of recommendations. Acad Med. 2014;89(9):1245-1251.

		Reporting Item	Page Number
**Title**			
	#1	Concise description of the nature and topic of the study identifying the study as qualitative or indicating the approach (e.g. ethnography, grounded theory) or data collection methods (e.g. interview, focus group) is recommended	1

For peer review only

Abstract

	#2	Summary of the key elements of the study using the abstract format of the intended publication; typically includes background, purpose, methods, results and conclusions	2

Introduction

Problem formulation	#3	Description and signifcance of the problem / phenomenon studied: review of relevant theory and empirical work; problem statement	3
Purpose or research question	#4	Purpose of the study and specific objectives or questions	3

Methods

Qualitative approach and research paradigm	#5	Qualitative approach (e.g. ethnography, grounded theory, case study, phenomenolgy, narrative research) and guiding theory if appropriate; identifying the research paradigm (e.g. postpositivist, constructivist / interpretivist) is also recommended; rationale. The rationale should briefly discuss the justification for choosing that theory, approach, method or technique rather than other options available; the assumptions and limitations implicit in those choices and how those choices influence study conclusions and transferability. As appropriate the rationale for several items might be	3

For peer review only

		discussed together.	
Researcher characteristics and reflexivity	#6	Researchers' characteristics that may influence the research, including personal attributes, qualifications / experience, relationship with participants, assumptions and / or presuppositions; potential or actual interaction between researchers' characteristics and the research questions, approach, methods, results and / or transferability	4,5
Context	#7	Setting / site and salient contextual factors; rationale	3
Sampling strategy	#8	How and why research participants, documents, or events were selected; criteria for deciding when no further sampling was necessary (e.g. sampling saturation); rationale	3,4
Ethical issues pertaining to human subjects	#9	Documentation of approval by an appropriate ethics review board and participant consent, or explanation for lack thereof; other confidentiality and data security issues	5
Data collection methods	#10	Types of data collected; details of data collection procedures including (as appropriate) start and stop dates of data collection and analysis, iterative process, triangulation of sources / methods, and modification of procedures in response to evolving study findings; rationale	6
Data collection	#11	Description of instruments (e.g. interview guides,	6

For peer review only

instruments and technologies		questionnaires) and devices (e.g. audio recorders) used for data collection; if / how the instruments(s) changed over the course of the study	
Units of study	#12	Number and relevant characteristics of participants, documents, or events included in the study; level of participation (could be reported in results)	5
Data processing	#13	Methods for processing data prior to and during analysis, including transcription, data entry, data management and security, verification of data integrity, data coding, and anonymisation / deidentification of excerpts	5
Data analysis	#14	Process by which inferences, themes, etc. were identified and developed, including the researchers involved in data analysis; usually references a specific paradigm or approach; rationale	5
Techniques to enhance trustworthiness	#15	Techniques to enhance trustworthiness and credibility of data analysis (e.g. member checking, audit trail, triangulation); rationale	5
**Results/findings**			
Syntheses and interpretation	#16	Main findings (e.g. interpretations, inferences, and themes); might include development of a theory or model, or integration with prior research or theory	6-11
Links to empirical data	#17	Evidence (e.g. quotes, field notes, text excerpts, photographs) to substantiate analytic findings	9-11

For peer review only

Discussion

Intergration with prior work, implications, transferability and contribution(s) to the field	#18	Short summary of main findings; explanation of how findings and conclusions connect to, support, elaborate on, or challenge conclusions of earlier scholarship; discussion of scope of application / generalizability; identification of unique contributions(s) to scholarship in a discipline or field	11-13
Limitations	#19	Trustworthiness and limitations of findings	11

Other

Conflicts of interest	#20	Potential sources of influence of perceived influence on study conduct and conclusions; how these were managed	13
Funding	#21	Sources of funding and other support; role of funders in data collection, interpretation and reporting	13

The SRQR checklist is distributed with permission of Wolters Kluwer © 2014 by the Association of American Medical Colleges. This checklist was completed on 31. July 2019 using https://www.goodreports.org/, a tool made by the EQUATOR Network in collaboration with Penelope.ai

For peer review only

Themes and illustrative quotes

Themes	Selected quotes
**Comprehension of the procedure varied considerably**	"I barely knew anything about episiotomy before the delivery."(#6, 28 years old, primipara, six months after childbirth)<br><br>“The doctors didn’t inform me about the procedure(episiotomy). After childbirth, the woman in the same delivery ward asked me ‘did you get episiotomy’ and I reply ‘what’s the episiotomy?’ I didn’t know it before and I finally realized what the anesthesia and suturing meant at that time.” (#9, 33 years old, primipara, four days after childbirth)<br><br>"I was ready to have a normal vaginal birth…When the day came, the baby was stuck because the perineal skin was very tight. [Therefore, I had an episiotomy.] I used to wonder what episiotomy is, and only came to know exactly what it is after childbirth. I basically had no idea about episiotomy before childbirth, and at that time I know it -- Oh, this is episiotomy!" (#14, 28 years old, primipara, one week after childbirth)<br><br>"I think it is necessary to do episiotomy when it can accelerate the progress of labor. But if the baby can be delivered smoothly, episiotomy should be avoided. After all, it is still a surgery." (#11, 30

	years old, primipara, two months after childbirth)<br><br>"The hospital takes episiotomy as a routine practice during normal vaginal birth. I think if episiotomy can relieve your suffering, routine episiotomy should be recommended. I felt that my perineum recovered soon after episiotomy. On the other hand, episiotomy won't cause any big problems, as long as you move carefully and clean yourself frequently." (#26, 28 years old, primipara, two weeks after childbirth)<br><br>“The doctor said that my uterine contractions were too weak, but I didn't feel that way. I just needed some time. I don't like the episiotomy at all. I searched episiotomy on the Internet and found its rate in China is excessively high. Many situations are not necessary. The doctors might be afraid of potential risks. I think if there is nothing wrong with the puerpera, the episiotomy should be avoided as far as possible. Previous generation like my mother didn’t use episiotomy but they recovered quite well." (#8, 34 years old, primipara, two months after childbirth)
**The pain and discomfort can interfere with daily life for weeks**	“I still feel pain of my perineal wound now and I can feel the difference between the two sides of perineum…the right side with the episiotomy lack skin elasticity. And there was an obvious scar in my perineum when I had the postpartum check-up at 42 days after childbirth.” (#1,

For peer review only - http://bmjopen.bmj.com/site/about/guidelines.xhtml

	35 years old, multipara, EP with forceps, six months after childbirth)<br><br>" When I tried to sit down, it seems like that I was sitting on a knife. The wound seemed to split. It hurt that much.” (#16, 32 years old, primipara, two years after childbirth)<br><br>"The wound hurt in the first few days. Five days after delivery, I started to feel better, but I can still feel the pulling or tugging pain at the incision…It was a bit tight." (#26, 28 years old, primipara, two weeks after childbirth)<br><br>“I had to move slowly because I felt painful when I moved suddenly. (#12, 40 years old, multipara, two months after childbirth)<br><br>"The doctor said I must sleep on the side. It was uncomfortable to sleep one-sided for a long time." (#6, 28 years old, primipara, six months after delivery)<br><br>"The healing was not very good [of my perineum], because the incision was relatively big. I went to emergency room twice within the first month [after childbirth]. The reason was the wound got infected. After removing the stiches, I was getting recovered from the cut. In the first few days, I was fed by my mother. I couldn't sit, and I just lay down there. I ate on the bed in the first month. " (#20, 30 years old, primipara, EP with forceps delivery, two months after childbirth)

For peer review only - http://bmjopen.bmj.com/site/about/guidelines.xhtml

	"My cut was six centimeters and it was done by a young midwife. The wound split at the six day after birth, then I suffered a lot because it recovered slowly. The pain had continued for half a month and the stiches cannot be absorbed …At that time, I couldn't sit or squat, and I had to move very slowly. Even now, I am still feeling painful when I am sitting" (#8, 34 years old, two months after childbirth)<br><br>"My wound hurt very much in the first week, and I couldn't peep or poop at all because I couldn't sit on the toilet. Every time using the toilet was like a torture to me. I think that most women who have received an episiotomy would probably have the same problem as me." (#1, 35 years old, multipara, EP with forceps, six months after childbirth)<br><br>I didn't want to use the toilet for bowel movement [defecation] until I had to poop…the fourth day after childbirth…The wound was so painful and I feared it would split when I exerted to poop…I also got hemorrhoids at that time, which became an obstacle of bowel movement. (#26, 28 years old, primipara, two weeks after childbirth)<br><br>"I just felt the wound painful during breastfeeding. Sometimes I felt that my baby couldn't get the nipples, so I just leaned forward a bit, and suddenly, the episiotomy wound began to hurt. After all, there were two

For peer review only - http://bmjopen.bmj.com/site/about/guidelines.xhtml

	cuts in my perineum [The women also got episiotomy at her last childbirth] …and I don't like to lie down [to feed the baby]." (#4, 34 years old, multipara, two months after childbirth)<br><br>"It was very tiring and painful to sit down… I felt my wound was also swollen, and I had to sit on one-side, lean my body to the side without episiotomy. I sat in this way for the breastfeeding within the whole first month …. This made my back hurt and sometimes it was really awful." (#28, 21 years old, primipara, six months after childbirth)<br><br>"…. If the wound gets infected because of improperly sterilization during the procedure, it would be very troublesome. The healing will take one to three weeks. In this kind of case, women with episiotomy would be more tortured than those with C-section." (Obstetrician, 28 years of relevant work experience, district MCH hospital.)<br><br>"Catgut (a kind of stitches) absorption varies from people to people, and some women may be allergic… have catgut rejection. If the sutures cannot be absorbed, it is always a foreign matter in perineum, which will hurt. " (Midwife, 25 years of relevant work experience, district MCH hospital)<br><br>"Episiotomy does have impacts on daily activities, such as breastfeeding. Some women are unwilling to breastfeed while lying

For peer review only

For peer review only - http://bmjopen.bmj.com/site/about/guidelines.xhtml

	down, or they just don't know how to breastfeed while lying down. Sometimes, people would feel anxious because of the pain. The milk secretion could also be affected by the pain." (Midwife, 20 years of relevant work experience，secondary general hospital)<br><br>"Walking and breastfeeding could be affected [by EP]. Episiotomy has a major impact on breastfeeding, because some women have short nipples, and it is difficult for the baby to suck the nipples when in lying position. Thus, it is easier to breastfeed with sitting position (Community health care provider, 17 years of relevant work experience, community health center)
**Long term physical and psychological effects can cause anxiety**	"We used the method of withdraw when having sex, because the inside of my perineum was uneven and rough, which hooked the condom and affected intercourse." (#13, 39 years old, primipara, seven years after childbirth)<br><br>"We returned to sexual life quite late, about half a year after delivery. The first three or four times [of sexual life] turned out painful because of the wound and vaginal dryness. When my baby was 10 months old, it wasn't painful anymore." (#23, 29 years old, primipara, 14 months after childbirth)<br><br>"Because of the terribly perineal pain, I asked my husband to resume

For peer review only - http://bmjopen.bmj.com/site/about/guidelines.xhtml

	sexual life a year later. I didn't dare to do it, because I worried the wound would pain again." (#16, 32 years old, primipara, two years after childbirth)<br><br>"Psychologically, I feel that the vagina cannot recover to original status…you feel the vagina is looser than before. And your spouse also has some psychological barriers to postpartum sexual life. I feel that many mothers who undergo episiotomy will have the shadows of sexual life more or less. The psychological shadow might disappear over time, but I don't know yet. (#1, 35 years old, multipara, six months after childbirth)<br><br>"The biggest problem for me now is the next delivery. Will I receive an episiotomy again? Will my second vaginal delivery encounter difficulties because of my rough perineum [the wound of episiotomy this time]?" (#3, 32 years old, primipara, EP with forceps, 14 months after childbirth)<br><br>" I don't dare to deliver my second child through normal birth (vaginal delivery). The experience of recovering from the episiotomy was indeed miserable. It really scared me. Maybe not having a second child is better… or maybe I would choose C-section even though it has some negative effects. …… if I had a vaginal birth again, and an

For peer review only - http://bmjopen.bmj.com/site/about/guidelines.xhtml

	episiotomy again. I cannot imagine what will happen, my vagina would be totally 'useless' for sexual life." (#13, 39 years old, primipara, two years after childbirth)<br><br>"The doctor directly did the episiotomy at my first childbirth. So I gained some childbirth experience and I was always afraid that I would suffer episiotomy again during this childbirth. There was a psychological shadow when I thought of the childbirth… I was worried about these problems such as deliver again, episiotomy again, miserable recovery of episiotomy. Finally, I still got episiotomy again!" (#29, 30 years old, multipara, six months after childbirth)<br><br>"Some women can't have intercourse at all. Once I met a case that the vagina of the women so tight that I can' even put one finger in, due to the scar contracture." (Obstetrician,28 years of relevant work experience, district MCH hospital)<br><br>"It doesn't have a lot of affection, because interval between births is generally long. It takes at least one year, right? The skin would recover within a year.…it's just that the wound will look ugly but the birth process won't be affected." (Midwife, 25 years of relevant work experience, district MCH hospital)<br><br>"Some people are scar physique (a kind of people who easily have

For peer review only - http://bmjopen.bmj.com/site/about/guidelines.xhtml

	enormous scar). This kind of scar is hard and protuberant so that we fear the wound would tear again during the second childbirth. If the scar tear and was sew up again, it can't heal very well. (Midwife, 25 years of work experience, secondary general hospital)<br><br>"What are the impacts of episiotomy on further childbirth? It is true that it may cast a psychological shadow on those women. If the episiotomy wound from first childbirth is infected or she had a severe tear, she won't dare to have another child, or she might choose C-section. Next time she might say: 'I don't want another baby' and 'I want to choose C-section instead of vaginal delivery."(Obstetrician, 28 years of relevant work experience, district MCH hospital)
**Social norms assume women will not complain of pain and should suffer alone**	"Whenever I said I felt sore of the perineal wound, they would say, 'why you still feel painful after 4 months?'. It sounds like I shouldn't be sore. Every time my husband said these words, I would response to him, 'you should get a cut and experience the healing process." (#28, 21 years old, primipara, six months after childbirth)<br><br>"I wondered if all the women would have the perineal pain after the childbirth…They [family members], such as my sister in law said that I was a bit low-tolerant, ...they all had birth experience but they never heard that a puerpera unable to sit down after childbirth. At that time, I

review only

	felt it was so hard to be a woman." (#16, 32 years old, primipara, two years after childbirth)<br><br>"No, I'm not very low-tolerant. Even if it hurts, I would endure the pain and not mention it. It didn't hurt that much. I could still bear with it. Some women are too spoiled to bear any pain and they always groan, which I thought it is meaningless. Nobody could replace your sufferings. It's normal thing, also the destiny of every woman." (#29, 30 years old, multipara, six months after childbirth)<br><br>“It was painful during suturing. It was the sharp tingling when I got the cut but the pain of the childbirth was more painful than this. After local anesthetic, it was still very painful and he sewed a few stitches. But I was immersed in the joy of having a baby at that time so I felt the pain could be tolerated…but I was trembling because of pain and the doctor comforted me ‘it would be finished soon, you can bear it, yes?” (#23, 29 years old, primipara, 14 months after delivery)<br><br>“I felt no pain about the cut because of the anesthetic…the suturing process was more painful. I cannot keep unmoved because the anesthetic effects tailed off later. And the doctor kept telling me not to move, saying that he couldn’t sew up well if I still move. But it was painful and he was sewing up for a long time because my wound was

For peer review only

For peer review only - http://bmjopen.bmj.com/site/about/guidelines.xhtml

	very big...I couldn't stay still, and I didn't know whether the stitches were done properly. I don't know if it related to my unabsorbed suturing knot, maybe it resulted from my own body condition (some immune factor). Doctors also can't change anything(sigh)" (#20, 30 years old, primipara, EP with forceps delivery, two months after delivery)<br><br>"I felt painful so much! I thought I really needed some treatments to relive the pain but the doctor thought I could endure this kind of pain……I really can't endure it since my wound is very large. I hadn't fallen asleep for several days after childbirth. The pain was so awful!" (#20, 30 years old, primipara, EP with forceps, two months after childbirth)

For peer review only

For peer review only - http://bmjopen.bmj.com/site/about/guidelines.xhtml

GRIPP2-SF reporting checklist for patient and public involvement

Section and topic	Item	Reported on page No
Aim	Report the aim of patient and public involvement in the study	Page 4
Methods	Provide a clear description of the methods used for patient and public involvement in the study. Please state if there was no patient and public involvement.	Page 4
Study results	Outcomes—Report the results of patient and public involvement in the study, including both positive and negative outcomes	Page 12
Discussion and conclusions	Outcomes—Comment on the extent to which PPI influenced the study overall. Describe positive and negative effects	Page 12
Reflections/critical perspective	Comment critically on the study, reflecting on the things concerning patient public involvement that went well and those that did not, so others can learn from this experience	Page 12

For peer review only

BMJ Open

Women's experience of episiotomy: a qualitative study from China

Journal:	*BMJ Open*
Manuscript ID	bmjopen-2019-033354.R1
Article Type:	Original research
Date Submitted by the Author:	11-Dec-2019

Complete List of Authors:	He, Siyuan; Fudan University, Maternal, Child and Adolescent Health, School of Public Health; Shanghai Municipal Center for Health Promotion<br>Jiang, Hong; Fudan University, Maternal, Child and Adolescent Health, School of Public Health<br>Qian, Xu; Fudan University, Maternal, Child and Adolescent Health, School of Public Health<br>Garner, Paul; Liverpool School of Tropical Medicine, Centre for Evidence Synthesis in Global Health, Department of Clinical Sciences

**Primary Subject Heading**:	Obstetrics and gynaecology
Secondary Subject Heading:	Sexual health, Qualitative research, Public health
Keywords:	QUALITATIVE RESEARCH, PRIMARY CARE, Community gynaecology < GYNAECOLOGY, GYNAECOLOGY

SCHOLARONE™
Manuscripts

I, the Submitting Author has the right to grant and does grant on behalf of all authors of the Work (as defined in the below author licence), an exclusive licence and/or a non-exclusive licence for contributions from authors who are: i) UK Crown employees; ii) where BMJ has agreed a CC-BY licence shall apply, and/or iii) in accordance with the terms applicable for US Federal Government officers or employees acting as part of their official duties; on a worldwide, perpetual, irrevocable, royalty-free basis to BMJ Publishing Group Ltd ("BMJ") its licensees and where the relevant Journal is co-owned by BMJ to the co-owners of the Journal, to publish the Work in this journal and any other BMJ products and to exploit all rights, as set out in our licence.

The Submitting Author accepts and understands that any supply made under these terms is made by BMJ to the Submitting Author unless you are acting as an employee on behalf of your employer or a postgraduate student of an affiliated institution which is paying any applicable article publishing charge ("APC") for Open Access articles. Where the Submitting Author wishes to make the Work available on an Open Access basis (and intends to pay the relevant APC), the terms of reuse of such Open Access shall be governed by a Creative Commons licence – details of these licences and which Creative Commons licence will apply to this Work are set out in our licence referred to above.

Other than as permitted in any relevant BMJ Author's Self Archiving Policies, I confirm this Work has not been accepted for publication elsewhere, is not being considered for publication elsewhere and does not duplicate material already published. I confirm all authors consent to publication of this Work and authorise the granting of this licence.

For peer review only

Title page

Title: Women's experience of episiotomy: a qualitative study from China

***Corresponding author:** Xu Qian, Mailbox 175, 138 Yixueyuan Road, Shanghai, 200032, China; email: xqian@shmu.edu.cn; Tel: +86 021 5427267

All co-authors:

#Siyuan He

Department of Maternal, Child and Adolescent Health, School of Public Health, Fudan University, Shanghai, China.

Shanghai Municipal Center for Health Promotion, Shanghai, China.

#Hong Jiang

Department of Maternal, Child and Adolescent Health, School of Public Health, Fudan University, Shanghai, China.

*Xu Qian

Department of Maternal, Child and Adolescent Health, School of Public Health, Fudan

For peer review only - http://bmjopen.bmj.com/site/about/guidelines.xhtml

University, Shanghai, China.

Paul Garner

Centre for Evidence Synthesis in Global Health, Department of Clinical Sciences,

Liverpool School of Tropical Medicine, Liverpool, UK.

SH and HJ contributed equally to this work.

Word count： 4156 words

For peer review only

For peer review only - http://bmjopen.bmj.com/site/about/guidelines.xhtml

ABSTRACT

Objectives To describe women's experience of episiotomy in urban China.

Design Semi-structured, in-depth interviews with women after episiotomy. We analyzed transcriptions using thematic analysis in Chinese. Emerging themes were debated in English to finalize interpretation.

Settings Two community health centers and four hospitals in Shanghai, China.

Participants Purposive sampling of 30 postpartum women who had experienced episiotomy; twenty-five were primiparous, and four had deliveries by forceps. We interviewed health providers to complement the data.

Results We identified four main themes: a) Women's knowledge of the procedure varies considerably; b) The pain interferes with daily life for weeks; c) Long term anxiety is a consequence for some, described as a "psychological shadow"; d) Societal norms assume women will not complain.

Conclusion Women receive little information in advance about episiotomy, yet the procedure has a wide range of physical and psychological consequences. This includes long term anxiety about the damage done to them as women.

Key words: episiotomy, perineal trauma, women's experience

Strengths and Limitations of this study

For peer review only - http://bmjopen.bmj.com/site/about/guidelines.xhtml

- This study was one of a few qualitative studies to explore women's experience of episiotomy after childbirth
- The study identified an effect of episiotomy described in Chinese as a "psychological shadow", and that societal norms meant women felt they were expected to suffer alone and not complain
- We interviewed women at different times after episiotomy, and were not able to evaluate whether their perceptions changed over time

For peer review only

For peer review only - http://bmjopen.bmj.com/site/about/guidelines.xhtml

INTRODUCTION

Doctors introduced episiotomy as a surgical procedure in the 1950's to reduce the risk of severe perineal tear, shorten delivery, and prevent damage to the pelvic floor.[1] However, the procedure can cause pain in the immediate postpartum period, the wound can become infected, and the scar can cause long-term dyspareunia. Indeed, the benefits of routine episiotomy have been contested.[2] This balance between benefits and harms has been evaluated in randomized controlled trials. These are summarized in the Cochrane review, and this shows that there is no evidence that routine episiotomy has the benefits originally assumed; and that more restricted use results in fewer women experiencing severe perineal or vaginal trauma.[3]

International institutions and professional societies now recommend episiotomy only when there is a clear clinical indication.[4-7] Practices in most European countries[8] show rates have fallen. However, episiotomy rates in vaginal births are still high in some countries; for example, 53.2% in Chile[9] (from hospital records), 73% from a hospital in Lebanon,[10] and 92% from a hospital in Cambodia.[11]

Pushback from consumers in the early 1990's may have contributed to the decline in routine episiotomy in the UK, but in general recommendations for episiotomy have been set up mainly from the health provider's medical standpoint, with little reference to the views or preferences of women. The Cochrane review (2017) pointed out that trials inadequately considered women's preferences, views on the procedures, or the outcomes that are important to them.[3]

For peer review only - http://bmjopen.bmj.com/site/about/guidelines.xhtml

More recently, the World Health Organization recognized the need for a “positive childbirth experience”,[4] which corresponds to the new Global Strategy for Women’s, Children’s and Adolescents’ Health (2016–2030). With an increase in emphasis to women-centered outcomes in clinical decision making, women’s experience of episiotomy is highly relevant.

In China, episiotomy used to be a routine practice for vaginal deliveriy.[12 13] In the last decade, hospital data reported levels of 47.4% to 84.7%;[14-17] and, some multi-center studies reported hospital rates from 41.2% to 69.7%.[18 19] For China, where there were 17.23 million births in 2016,[20] there could be as many as 7.33 million episiotomies a year (given a vaginal birth rate of 61.0% and episiotomy rate of 69.7% among vaginal birth).[18] Although the Chinese national obstetric guideline has recommended restrictive use of episiotomy since 2016, it has not been implemented.[21]

We found no data in the published English literature on the experience of episiotomy in women in China, and therefore carried out this study. Through this qualitative study, we aimed to describe how women experience in urban China.

METHODS

Approach, setting and sampling strategy

We used standard qualitative methods with semi-structured, in-depth individual interviews. The details of the methods were reported according to the SRQR reporting

For peer review only - http://bmjopen.bmj.com/site/about/guidelines.xhtml

checklist (see Supplementary File 1).[22] We conducted the study in Shanghai (population 24.2 million (2016)). The city has had a policy of routine episiotomy from 1999; and the rate around 35.8% to 86.67% from 2011 to 2014.[23-25] Community health centers hold pregnancy registration and information to allow home visits in the postnatal period for clients in their catchment area, while clinical services for childbirth are provided by higher level of hospitals, and episiotomy practices vary between hospitals. Compared with general hospitals, maternal and child health (MCH) hospitals are more likely to adopt restrictive episiotomy policy since their midwives are experienced and well trained, and women at these settings tend to be low-risk. We used the two community health centers where we had worked previously and thus staff were familiar with us: one in Pudong District, east of Shanghai (1459 pregnant women registered in 2017); and one in Xuhui District, west of Shanghai (775 pregnant women registered in 2017).

We used purposive sampling strategy, seeking women over 18 years old who had undergone an episiotomy in her last birth. We recruited women from three different postpartum periods (within two weeks, no more than six months and six months above after childbirth); we also took account of the types of hospitals to ensure a mix of experiences. Women being invited for this study delivered in various types of hospitals including municipal MCH hospital, tertiary general hospital, district MCH hospital and secondary general hospital. Experienced health care providers who had over three years work experience in maternal health area were recruited to confirm women's symptoms and help to better understand women's views and reflections. Two or three health care

For peer review only - http://bmjopen.bmj.com/site/about/guidelines.xhtml

providers from each type of hospitals were involved in this study and their characteristics are shown in the Supplementary File 2. We stopped interviewing women when we appeared not to identify new information.[26-28] The primary researcher (SH) carried out the interviews, under the guidance of the supervisors (HJ and XQ). SH is a master student, who had received training in qualitative methods and had a six-month work placement with the MCH administration.

Ethical approval

All participants were informed about the research purpose and contents. Interviews were conducted after written informed consent obtained from each participant. The research obtained the approval from the Institutional Review Board in School of Public Health, Fudan University.

Patient and public involvement

When designing the study, we invited a few women to give us feedback on the approach and the questions to ask. We collected women's comments on the public internet forum and interviewed four women about the research topic before we designed the interview guide. This preliminary work led to several revisions to the interview guide.

Data collection

We approached women by accompanying health staffs during postpartum home visits or when women brought their children for child health checkup in community health centers

For peer review only

For peer review only - http://bmjopen.bmj.com/site/about/guidelines.xhtml

between September 2017 and March 2018. We used an interview guide based on the literatures and our research group discussions (Table 1);[29-31] also, we reviewed women's comments on the public internet forum to improve the design of interview guide and piloted the interview guide with four postpartum women. The piloted data were also included in our analysis as it was consistent with the main sample. Interviews were conducted in private rooms in the community health centers, hospitals and interviewees' homes and all women provided signed consents. Interviews were in Chinese and recorded with permission. For the health providers, they were recommended by relevant administrators and invited to this study. They were interviewed at a private room in their workplaces.

Table 1 Interview guide

What's your experience after episiotomy, from the childbirth to postpartum period? (Probe: discomfort, pain, swelling)
Did episiotomy impact on your daily life? How? (Probe: walking, sitting, breastfeeding, baby care, sexual life, medication, mood)
How did you deal with your suffering or problems? (Probe: medical services usage)
Are there some long-lasting effects of episiotomy you have noticed? If yes, what are they?

Data analysis

For peer review only - http://bmjopen.bmj.com/site/about/guidelines.xhtml

Medical master students transcribed interviews, and one of the interviewers (SH) checked them for accuracy. We used NVivo8.0 (QSR) software for thematic analysis.[32 33] Two researchers (SH and YC) read all the transcripts and coded the data to identify the reoccurring topics, ideas, or concepts independently. After discussing the differences of the coding, they organized the data into initial themes. Initial themes and quotes were translated into English and checked by XQ. All the co-authors then further reflected on these themes and developed overarching categories, discussing the themes in both Chinese and English.

We constructed tables that included quotes that substantiated themes. This table makes clear how the themes were developed and helps us convey important substantiated messages within a specific word limit. The health professionals' responses were grouped against the emergent themes from the women's interviews and included within each theme.

During this process, on two occasions we found themes that we could not translate directly into English. Rather than being a problem, these were both informative and underlying themes. The team discussed the words carefully in Chinese and English to gain a common understanding of meaning and cultural context.

The research team included three bilingual speakers (SH, HJ, XQ) and one native English speaker (PG). All the themes, descriptions and corresponding quotes were checked by all the authors.

For peer review only - http://bmjopen.bmj.com/site/about/guidelines.xhtml

Reflexivity

As a team, we discussed our prior beliefs and experiences in early discussions and during analysis to reflect on how this may influence our analysis. The research team included people that had performed, repaired and experienced episiotomy (HJ, QX, PG). Evaluating episiotomy and the uncertainty around benefits and harms is a topic of interest to all the authors, and, as with many medical and obstetrical interventions, we as researchers remain "healthy sceptics". Three authors have completed the Cochrane review examining this topic (HJ, QX, PG) and reporting is that consumer views on the procedure are important for medical policy. All had experience in collecting and analyzing qualitative data; PG and QX have worked together for over 20 years on projects about whether obstetric practice and research evidence are in alignment in China.

RESULTS

We interviewed 30 postpartum women, age range from 21 to 40 (mean age 30.1) years. Twenty-five women were primiparous; all had experienced episiotomy and four also received assisted delivery with forceps. Seven women were interviewed within two weeks, nine were at two months, and fourteen were above six months after childbirth (Table 2).

Four main themes emerged and the selected quotes of each theme were shown at the end of the results (Table 3). The complete illustrative quotes were shown in the

For peer review only - http://bmjopen.bmj.com/site/about/guidelines.xhtml

Supplementary File 3.

For peer review only

For peer review only - http://bmjopen.bmj.com/site/about/guidelines.xhtml

Table 2 Characteristics of the Postpartum Women

	Women
**Age (year)**	
Mean±SD	30.1±3.8
Range	21~40
**Parity**	
Primipara	25
Multipara	5
**Mode of delivery**	
Episiotomy	26
Episiotomy with forceps	4
**Interview time**	
Within two weeks after childbirth	7
No more than six months after childbirth	9
Six months above after childbirth	14

For peer review only

Women's knowledge of the procedure varies considerably

Nearly one fifth of women knew very little about the procedure before childbirth. For some, their understanding of episiotomy was shaped by their experiences in real time during delivery, and the people around them. For example, one woman heard about it from the woman being wheeled out of the delivery ward; another woman had heard the word, but was not clear what it meant, until it happened: "The doctor cut me and then I

For peer review only - http://bmjopen.bmj.com/site/about/guidelines.xhtml

thought, Yes, now I know what it is!" Besides these uninformed women, more than one third of women simply knew that "episiotomy is a cut to the vagina" and stated it sounded frightening but knew little more.

A few women seemed to be more informed, from a variety of sources: online resources, discussion with other women, and from doctors. These more informed women were able to express the concept of balancing benefits and harms in their conversations: they described fear of the procedure and the damage to the vagina, but accepted that the procedure would accelerate the progress of labor, guarantee the safety of the baby, and avoid tears.

Women's personal experience was, unsurprisingly, important in shaping their views: one woman accepted routine episiotomy was required, and another multipara who had an episiotomy with her first childbirth requested it for her second delivery. These women had few problems with their current procedures appeared to accept the need for the procedure. However, those who had a miserable experience seemed more likely to complain the negative effects and question the need for an episiotomy.

The pain interferes with daily life for weeks

Women's pain and discomfort varied-but in some was severe, and in a few lasted for months. Women in pain for two weeks only described the pain as "a little pain or discomfort", but a few women reported considerable pain for months after childbirth, three reporting this as "intolerable" for more than one month. These women with severe

For peer review only - http://bmjopen.bmj.com/site/about/guidelines.xhtml

pain also reported problems with suturing including tight stitches, irritation from the stitches or the wound gaping.

Avoiding pain, and fear that the episiotomy would split, meant women avoided moving around. Women stated they were conscious of the wound and avoiding pain, so had to walk or move slowly, or avoid contact as the wound hurt when pressed. Some had to sit or lie on one-side or stay in one position for a long time to avoid pain, and this made them tired and uncomfortable. Three women with problems with the episiotomy healing complained that they could not sit down for a minute because of the horrible pain, which made them incapable of looking after their babies and themselves.

For those with pain, several volunteered this interfered breastfeeding, and most women believed the wound of episiotomy affected breastfeeding somehow. Since women liked to feed baby whilst sitting, if this was painful then they struggled to feed. Some of them learned to breastfeed by lying down or using breast pump in a standing position. Other women sat in pain and found it a struggle, increasing the difficulty and fatigue of breastfeeding.

The pain often interfered with defecation, with increasing pain and the sensation of the wound about to split whilst defecating. Just sitting or squatting was also hard. This fear of pain or that the wound would split open led women to avoid defecation, worsening existing postpartum constipation. Two women even mistakenly regarded the pain of episiotomy as the pain of hemorrhoids for weeks.

For peer review only - http://bmjopen.bmj.com/site/about/guidelines.xhtml

Health providers had different views on the women’s experience. They considered that perineal pain from episiotomy is usually tolerable and does not last long, unless there is something wrong such as infection or stitches that could not be absorbed. The doctors did not mention the effects with breastfeeding. On the other hand, the community health care providers and midwives who confirmed the difficulties of breastfeeding. Some commented that some women had to breastfeed in a painful sitting position because they didn’t know how to feed baby in any other way; and that the sitting posture was the proper way for the baby to suck mother’s nipples. Postpartum constipation and pain of defecation were all recognized as problems by the health professionals.

For peer review only

For peer review only - http://bmjopen.bmj.com/site/about/guidelines.xhtml

Long term anxiety is a consequence for some, described as a "psychological shadow"

Several women used the word "psychological shadow" cast by the long-term effects from episiotomy. The Chinese word implies a negative experience of suffering or torment that leads to a dread or worries of the future-a bit like the experience of war or a tumultuous personal event. These women were vocal and had much to say about the effect on them: they were anxious about what would happen when they had sex, or if they were to have another baby.

The concern about sex was also apparent in those women who resumed sex: they mentioned the uneven or rough skin of perineal wound and painful intercourse, which affected the enjoyment of sexual life. One woman said the pain with sex might have arisen from her anxiety-the psychological shadow. Another woman asked her husband to await till one year after childbirth, because she suffered severe pain of episiotomy for nearly two months and feared sexual life might take her back to the nightmare again. Some responses around resumption of sex and the "psychological shadow" included beliefs that their vagina was damaged and loose and may not ever recover. For these women, they were unwilling to resume sexual life e and described being permanently "changed" that there had been damage done to their vagina.

The "psychological shadow" also impacted on how women viewed a possible subsequent pregnancy. Most expressed concern as to whether the episiotomy wound would hinder the process of next vaginal delivery; whether the wound would split again in the next vaginal delivery; or whether they would be subject to another episiotomy. This influenced

For peer review only - http://bmjopen.bmj.com/site/about/guidelines.xhtml

how they viewed subsequent pregnancies: at least one woman reported that next time she would ask for a cesarean section to avoid episiotomy. One woman said, "if I had a vaginal birth again, and an episiotomy again. I cannot imagine what will happen, my vagina would be totally 'useless' for sexual life." The interviews indicated a high degree of anxiety about the long-term physical consequences and reflect how this then itself causes further anxiety. Several multiparous women who experienced episiotomy twice reported that they need longer time to recover from the repeated episiotomy.

The health providers were aware of physical abnormalities following episiotomy and psychological concerns about sexual life; most dismissed concerns about subsequent deliveries: one midwife thought the hard scar is easy to tear again if episiotomy isn't done in advance. However, most of them conceded that women who have a miserable experience of episiotomy can cause women's fear and anxiety over the next delivery.

For peer review only - http://bmjopen.bmj.com/site/about/guidelines.xhtml

Societal norms assume women will not complain

Women reported that their family members and health professionals regarded pain and discomfort as a normal part of childbirth and the puerperium. This pain and discomfort are expected and will gradually disappear without treatment. Whilst women accepted this, it appeared that this expectation did not take into account the more substantive pain, discomfort and interference with daily life associated with episiotomy (see our first theme) and this is distressing for women. Three women felt the expectations that they should not complain much about the “slight and temporary discomforts” but to be strong and endure the pain or discomfort by themselves.

Several women mentioned their family members expected them to endure the “a non-severe discomfort”. Indeed, two of them were frightened that if they complained too much they would be judged as “being low-tolerant” (Jiao qi). This word is a pejorative personality trait, which means a person exaggerate something that is slightly uncomfortable. This word refers to people who have “weak minds” and who are rather cowardly. When these two women expressed or complained the postpartum suffering too much, their families thought their discomforts were slight, that the women should have endured this, and were at risk of this weak character trait of being “low-tolerant”. One woman said her families thought the woman was exaggerating her pain when she said could not sit: they had also experienced normal birth and didn’t encounter anything that terrible. When these types of judgement happened, the women felt upset and unwilling to speak out, suffering alone. Surprisingly, another interviewed woman even regarded the

For peer review only - http://bmjopen.bmj.com/site/about/guidelines.xhtml

tolerance of pain as women’s own destiny. Thus, the societal norms make some women “suffer alone” and stop them from seeking help.

Indeed, these societal norms about tolerating pain also manifest in the way health care was provided. Most women thought suturing is more painful than being cut; yet some doctors did not check whether women were effectively anaesthetized during the suturing. One woman complained of pain during suturing but was told to “wait-it will be finished soon”; and another was told to stay still. One woman reported the pain was so severe she did move when being sutured, and then blamed herself for the subsequent healing problems because she had moved. The expected tolerance of pain extended to pain relief: a woman asked for pain relief after childbirth but was refused by the doctors with the reason “the level of pain after vaginal birth can be tolerated”.

For peer review only - http://bmjopen.bmj.com/site/about/guidelines.xhtml

Table 3 Summary of themes with selected quotes

Themes	Selected quotes
Women's knowledge of the procedure varies considerably	" The doctors didn't inform me about the procedure(episiotomy). After childbirth, the woman in the same delivery ward asked me 'did you get episiotomy' and I reply 'what's the episiotomy?' I didn't know it before and I finally realized what the anesthesia and suturing meant at that time." (#9, 33 years old, primipara, four days after childbirth)<br><br>"I think it is necessary to do episiotomy when it can accelerate the progress of labor. But if the baby can be delivered smoothly, episiotomy should be avoided. After all, it is still a surgery." (#11, 30 years old, primipara, two months after childbirth)<br><br>"The doctor said that my uterine contractions were too weak, but I didn't feel that way. I just needed some time. I don't like the episiotomy at all. I searched episiotomy on the Internet and found its rate in China is excessively high. Many situations are not necessary. The doctors might be afraid of potential risks. I think if there is nothing wrong with the puerpera, the episiotomy should be avoided as far as possible. Previous generation like my mother didn't use episiotomy but they recovered quite well." (#8, 34 years old, primipara, two months after childbirth)
The pain	"The healing was not very good [of my perineum], because the incision was

For peer review only - http://bmjopen.bmj.com/site/about/guidelines.xhtml

**interferes with daily life for weeks**	relatively big. I went to emergency room twice within the first month [after childbirth]. The reason was the wound got infected. After removing the stiches, I was getting recovered from the cut. In the first few days, I was fed by my mother. I couldn't sit, and I just lay down there. I ate on the bed in the first month. " (#20, 30 years old, primipara, EP with forceps delivery, two months after childbirth)<br><br>"It was very tiring and painful to sit down… I felt my wound was also swollen, and I had to sit on one-side, lean my body to the side without episiotomy. I sat in this way for the breastfeeding within the whole first month …. This made my back hurt and sometimes it was really awful." (#28, 21 years old, primipara, six months after childbirth)<br><br>"…. If the wound gets infected because of improperly sterilization during the procedure, it would be very troublesome. The healing will take one to three weeks. In this kind of case, women with episiotomy would be more tortured than those with C-section." (Obstetrician, 28 years of relevant work experience, district MCH hospital.)<br><br>"Episiotomy does have impacts on daily activities, such as breastfeeding. Some women are unwilling to breastfeed while lying down, or they just don't know how to breastfeed while lying down. Sometimes, people would feel anxious because of the pain. The milk secretion could also be affected by the

For peer review only - http://bmjopen.bmj.com/site/about/guidelines.xhtml

	pain." (Midwife, 20 years of relevant work experience , secondary general hospital)
**Long term anxiety is a consequence for some, described as a "psychological shadow"**	"We used the method of withdraw when having sex, because the inside of my perineum was uneven and rough, which hooked the condom and affected intercourse." (#13, 39 years old, primipara, seven years after childbirth)<br><br>"Because of the terribly perineal pain, I asked my husband to resume sexual life a year later. I didn't dare to do it, because I worried the wound would pain again." (#16, 32 years old, primipara, two years after childbirth)<br><br>" I don't dare to deliver my second child through normal birth (vaginal delivery). The experience of recovering from the episiotomy was indeed miserable. It really scared me. Maybe not having a second child is better… or maybe I would choose C-section even though it has some negative effects. …… if I had a vaginal birth again, and an episiotomy again. I cannot imagine what will happen, my vagina would be totally 'useless' for sexual life." (#13, 39 years old, primipara, two years after childbirth)<br><br>"It doesn't have a lot of affection, because interval between births is generally long. It takes at least one year, right? The skin would recover within a year.…it's just that the wound will look ugly but the birth process won't be affected." (Midwife, 25 years of relevant work experience, district MCH hospital)

For peer review only - http://bmjopen.bmj.com/site/about/guidelines.xhtml

	"Some people are scar physique (a kind of people who easily have enormous scar). This kind of scar is hard and protuberant so that we fear the wound would tear again during the second childbirth. If the scar tear and was sew up again, it can't heal very well. (Midwife, 25 years of work experience, secondary general hospital)
**Societal norms assume women will not complain**	"I wondered if all the women would have the perineal pain after the childbirth…They [family members], such as my sister in law said that I was a bit low-tolerant, ...they all had birth experience but they never heard that a puerpera unable to sit down after childbirth. At that time, I felt it was so hard to be a woman." (#16, 32 years old, primipara, two years after childbirth)<br><br>"No, I'm not very low-tolerant. Even if it hurts, I would endure the pain and not mention it. It didn't hurt that much. I could still bear with it. Some women are too spoiled to bear any pain and they always groan, which I thought it is meaningless. Nobody could replace your suffering. It's normal thing, also the destiny of every woman." (#29, 30 years old, multipara, six months after childbirth)<br><br>"It was painful during suturing. It was the sharp tingling when I got the cut but the pain of the childbirth was more painful than this. After local anesthetic, it was still very painful and he sewed a few stitches. But I was immersed in the joy of having a baby at that time so I felt the pain could be tolerated…but I was

For peer review only - http://bmjopen.bmj.com/site/about/guidelines.xhtml

	trembling because of pain and the doctor comforted me "it would be finished soon, you can bear it, yes?" (#23, 29 years old, primipara, 14 months after delivery)<br><br>"I felt painful so much! I thought I really needed some treatments to relive the pain but the doctor thought I could endure this kind of pain……I really can't endure it since my wound is very large. I hadn't fallen asleep for several days after childbirth. The pain was so awful!" (#20, 30 years old, primipara, EP with forceps, two months after childbirth)

For peer review only

DISCUSSION

There are few qualitative studies of episiotomy worldwide, and the ones we have identified do not differentiate between episiotomy alone and perineal trauma (including episiotomy and severe tear). Most qualitative studies focused on episiotomy only were usually conducted in hospital settings and concerned with shorter term consequences of episiotomy.[34-37] By contrast, we identified more information about women's perspectives and personal reflections in the community settings. The description of the "psychological shadow" seems an apt way to describe both the physical and psychological consequences, and how these play out together-for example with dyspareunia, where anxiety may worsen the physical experience.

The limitation of our study included, first, some women were interviewed more than six

For peer review only - http://bmjopen.bmj.com/site/about/guidelines.xhtml

months after childbirth, which might introduce the recall bias for their experiences shortly after episiotomy. Second, we interviewed the women at the different timelines after childbirth instead of performing at the three specified timelines for all participants, so we cannot know the duration of some women's suffering and the shift of their understanding of episiotomy.

When we set out, we anticipated the health care providers would validate women's perceptions, but they seemed unaware of the long-term consequences, and tended to underestimate the degree of pain and restricted function that women reported.

Episiotomy results in extensive physical discomfort for some, and life troubles to solve. In this study, we confirmed women did suffer the perineal pain or discomfort, consistent with qualitative and quantitative studies.[31 38 39] In addition, women in this study complained more about the unabsorbed stitches or split of stitches. Many reviews also indicate there are a part of women need removal or re-suture services due to factors of materials or skills.[40 41] Women in this study also reported episiotomy limited postpartum daily activities including sitting, breastfeeding, defecation, and intercourse.

We have found few studies reporting that episiotomy interferes with breastfeeding. Chou mentioned the perineal pain can interfere the initiation of breastfeeding,[42] and Persico found the exclusive breastfeeding rate of women with episiotomy in first day after delivery was lower than the women with intact perineum.[43] These physical symptoms or morbidity can also cause psychological burden or anxiety. A study in Jordan reported there was an association between post-partum depression and 15 health problems of obstetric,

For peer review only

gynecologic (that is, episiotomy pain, infection), and general health conditions (including fatigue and headache).[44] These physical problems might have cumulative effects, as a prospective study indicated high burden of breastfeeding problems alone or with co-morbid physical problems was associated with poor maternal mood at 8 weeks, while the high burden of physical health problems was not significantly associated.[45]

The influence on mood also may relate to sexual life, further delivery, and the impact on sex has been reported elsewhere.[46 47] Our findings highlighted that some women with episiotomy feared, or wanted to avoid another pregnancy because of the pain they experienced, or that they would choose C-section in the next childbirth. This is consistent with other qualitative studies about vaginal childbirth,[48 49] and a study from Turkey also indicated fear about impending childbirth can increase the likelihood of requesting a caesarean section.[50]

Episiotomy was administered in this study with women not even knowing it was going to happen. This lack of informed consent appears widespread and is reported in other studies. One study in Brazil mentioned half of interviewed women did not receive any information about the procedure before or during childbirth.[35] Another study reported that the procedure was informed but lack of authorization or was even practiced directly without any explaination.[36] Some women even did not know whether an episiotomy or spontaneous tear was done, and only noticed a greater discomfort during suturing.[34] Women particularly lacked the knowledges about the consequences of episiotomy in our study, one qualitative study about perineal trauma also identified the similar theme “being

For peer review only - http://bmjopen.bmj.com/site/about/guidelines.xhtml

unaware of the episiotomy's consequences".[35]

Women, their families, and even some health professionals in this region also showed little understanding of some of the possible consequences of episiotomy. This opinion is consistent with a systematic mixed studies review about perineal trauma reported the theme "normalization and feeling dismissed", which means women's health problems are regarded as a normal consequence after childbirth and their questions keep unanswered by health professionals.[51] Some studies reported women felt frustrated and abandoned because of the "dismissed by health care providers".[52 53]

The study also raised the interplay between physical injury and pain, the societal expectations that this was normal, and the women's personal anxieties about the anticipated damage to their genitalia and anticipated pain with sex. When a woman with both physical pain and anxiety are not expected to complain, this can make matters worse. These factors and interactions are particularly important in China where the episiotomy rates remain high.

CONCLUSION

Women were inadequately informed about episiotomy, but experienced consequences of the procedure, including pain, interference with daily life. These were compounded by social norms that expect them not to complain and longer-term anxiety about the physical and psychological effects on them as women.

For peer review only - http://bmjopen.bmj.com/site/about/guidelines.xhtml

Acknowledgments The authors thank the valuable contribution of the women and health care providers who participated in this study. The authors are very grateful to Qinjin Huang for her arrangements of study settings. The authors would like to thank Chunyi Gu and Yao Chen for the professional guidance and data analysis. The authors would also like to thank the following health staffs for their assistance of recruitment: Xiaoxing Dai, Jiali Zhang, Hongxia Cui, Linlin Li.

Author Contributions SH, HJ and XQ designed the study and analyzed data. SH and HJ drafted the paper, and XQ revised it. PG helped with analysis, commented on interpretation and helped write the manuscript. SH and HJ contributed equally to this study, who should be regarded as co-first authors. All authors have verified and approved the final version of the abstract for publication.

Competing interests None declared.

Funding This work received support from the Effective Health care Research Consortium, funded by UK aid from the UK government for the benefit of developing countries (Grant: 5242)

Details of ethics approval All participants were informed about the research purpose and contents. Interviews were conducted after written informed consent obtained from each participant. The research obtained the approval from the Institutional Review Board in School of Public Health, Fudan University (ID: 2017-12-0648)

For peer review only - http://bmjopen.bmj.com/site/about/guidelines.xhtml

Provenance and peer review Not commissioned; externally peer reviewed.

Data sharing statement Data are available upon reasonable request.

Open access This is an open access article distributed in accordance with the Creative Commons Attribution Non Commercial (CC BY-NC 4.0) license, which permits others to distribute, remix, adapt, build upon this work non-commercially, and license their derivative works on different terms, provided the original work is properly cited, appropriate credit is given, any changes made indicated, and the use is non-commercial. See: http:// creative commons. org/licenses/by-nc/4.0/.

References:

1. Longo LD. Classic pages in obstetrics and gynecology. A treatise of midwifery. In three parts by Sir Fielding Ould, Dublin, O. Nelson & C. Connor, 1742. *Am J Obstet Gynecol* 1976;124:656.
2. Muhleman MA, Aly I, Walters A, et al. To cut or not to cut, that is the question: A review of the anatomy, the technique, risks, and benefits of an episiotomy. *Clin Anat* 2017;30:362-72.
3. Jiang H, Qian X, Carroli G, et al. Selective versus routine use of episiotomy for vaginal birth. *Cochrane Database Syst Re* 2017;8:2: CD000081.
4. WHO recommendations: intrapartum care for a positive childbirth experience. Geneva: World Health Organization; 2018;150-155.
5. Overview | Intrapartum care for healthy women and babies | Guidance | NICE. Avaulable at: https://www.nice.org.uk/guidance/cg190. (Accessed 13 Jul 2019).
6. ACOG. ACOG Practice Bulletin. Episiotomy. Clinical Management Guidelines for Obstetrician-Gynecologists. Number 71, April 2006. *Obstet Gynecol* 2006;107:957-62.
7. RCOG. The Management of Third- and Fourth-Degree Perineal Tears. Green-top Guideline No. 29.(June. 2015).
8. Blondel B, Alexander S, Bjarnadottir RI, et al. Variations in rates of severe perineal tears and episiotomies in 20 European countries: a study based on routine national data in Euro-Peristat Project. *Acta Obstet Gynecol Scand* 2016;95:746-54.
9. Binfa L, Pantoja L, Ortiz J, et al. Midwifery practice and maternity services: A multisite descriptive study in Latin America and the Caribbean. *Midwifery* 2016;40:218-25.

For peer review only - http://bmjopen.bmj.com/site/about/guidelines.xhtml

10. Kaddoura R, DeJong J, Zurayk H, et al. Episiotomy practice in the Middle East: A Lebanese teaching tertiary care center experience. *Women Birth* 2019;32:223-228.

11. Schantz C, Sim KL, Ly EM , et al. Reasons for routine episiotomy: A mixed-methods study in a large maternity hospital in Phnom Penh, Cambodia. *Reprod Health Matters* 2015;23:68-77.

12. Qian X, Smith H, Zhou L, et al. Evidence-based obstetrics in four hospitals in China: An observational study to explore clinical practice, women's preferences and provider's views. *BMC Pregnancy Childbirth* 2001, 1(1):1.

13. Qian X, Smith H, Liang, J, et al. Evidence-informed obstetric practice during normal birth in China: trends and influences in four hospitals. *BMC Health Serv Res* 2006; 6: 29

14. Wang Q, Zheng S, Ni Y, et al. The effect of labor epidural analgesia on maternal - fetal outcomes: a retrospective cohort study. *Arch Gynecol Obstet* 2018;298:89-96.

15. Tung CW, Cheon WC, Tong WM, et al. Incidence and Risk Factors of Obstetric Anal Sphincter Injuries after Various Modes of Vaginal Deliveries in Chinese Women. *Chin Med J (Engl)* 2015;128:2420-5.

16. Yang X, Zhang HX, Yu HY, et al. The prevalence of fecal incontinence and urinary incontinence in primiparous postpartum Chinese women. *Eur J Obstet Gynecol Reprod Biol* 2010;152:214-7.

17. Zhao Y, Xiao M, Tang F, et al. The effect of water immersion delivery on the strength of pelvic floor muscle and pelvic floor disorders during postpartum period. *Medicine (Baltimore)* 2017;96:8124.

18. Zhu L, Li L, Lang J, et al. Prevalence and risk factors for peri- and postpartum urinary incontinence in primiparous women in China: a prospective longitudinal study. *Int Urogynecol J* 2012;23:563-72.

19. Liang L, Min L, Li N. [Survey on episiotomy among puerperal women in vaginal delivery from 2011 to 2014]. *Maternal and Child Health care of China* 2015;07:1087-9.

20. http://www.stats.gov.cn/tjsj/zxfb/201908/t20190822_1692898.html. (Accessed 22 Nov 2019).

21. Obstetrics Group, Obstetrics and Gynecology Society, Chinese Medical Association. [Clinical guidelines for operative vaginal delivery (2016)]. *Chin J Obstet Gynecol* 2016;51:565-7.

22. O'Brien BC, Harris IB, Beckman TJ, et al. Standards for reporting qualitative research: a synthesis of recommendations. *Acad Med* 2014, 89:1245-51.

23. Yang W. [Status of intrapartum services on evidence-based practice in Pudong Maternal and Child Health Hospital]. Fudan university 2013.

24. Ye Q，Niu L，Zhao L.[Investigation of Puerpera's Pelvic Floor Function and Early Effect of Obstetric Factors on Pelvic Floor Function]. *Journal of Practical Obstetrics and Gynecology* 2016;32:843-6

25. Zhuang W, Li Y, Wu N, et al. [Effects of Midwife Hierarchical Management mode on Improving the Quality Control of the Delivery Room]. *Nurs J Chin PLA* 2013; 30:52-4.

For peer review only - http://bmjopen.bmj.com/site/about/guidelines.xhtml

26. Glaser BAAS. The discovery of grounded theory: Strategies for qualitative research. New York: Aldine Publishing Company 1967

27. Guest G, Bunce A, Johnson L. How Many Interviews Are Enough? *Field Method* 2016;18(1):59-82.

28. Collumbien M, Busza J, Cleland J, et al. Social science methods for research on sexual and reproductive health. Malta: World Health Organization 2012.

29. Tully KP, Stuebe AM, Verbiest SB. The fourth trimester: a critical transition period with unmet maternal health needs. *Am J Obstet Gynecol* 2017;217:37-41.

30. Fahey JO, Shenassa E. Understanding and Meeting the Needs of Women in the Postpartum Period: The Perinatal Maternal Health Promotion Model. *J Midwifery Womens Health* 2013;58:613-21.

31. Dudley L, Kettle C, Waterfield J, et al. Perineal resuturing versus expectant management following vaginal delivery complicated by a dehisced wound (PREVIEW): a nested qualitative study. *BMJ Open* 2017;7:e13008.

32. Burnard P, Gill P, Stewart K, et al. Analysing and presenting qualitative data. *Br Dent J* 2008;204:429-32.

33. Themes and codes. In: Guest, G., MacQueen, K. M., & Namey, E. E. Applied thematic analysis. Thousand Oaks, CA: SAGE Publications 2012:49-78

34. Wey CY, Salim NR, Junior HPDO, et al. The practice of episiotomy: a qualitative study on perceptions of a group of women. *Online Brazilian Journal of Nursing* 2011;10;2. http://www.objnursing.uff.br/index.php/nursing/rt/printerFriendly/3332/html_2

35. Dengo VAR, Dos Santos Silva R, Souza SRRK, et al. Puerperal women's perceptions about episiotomy. *Cogitare Enferm* 2016;21:1-8.

36. Figueiredo G, Barbieri M, Gabrielloni MC, et al. Episiotomy: perceptions from adolescent puerperae. *Invest Educ Enferm* 2015;33:365-73.

37. Previatti JF, de Souza KV. [Episiotomy: focusing women's view]. *Rev Bras Enferm* 2007;60:197-201.

38. Gün İ, Doğan B, Özdamar Ö. Long- and short-term complications of episiotomy. *Turk J Obstet Gynecol* 2016;13:144-8.

39. Manresa M, Pereda A, Bataller E, et al. Incidence of perineal pain and dyspareunia following spontaneous vaginal birth: a systematic review and meta-analysis. *Int Urogynecol J* 2019;30:853-868.

40. Kettle C, Dowswell T, Ismail KM. Continuous and interrupted suturing techniques for repair of episiotomy or second-degree tears. *Cochrane Database Syst Rev* 2012;11: CD000947.

41. Selo-Ojeme DO, Okonkwo CA, Atuanya C, et al. Single-knot versus multiple-knot technique of perineal repair: a randomised controlled trial. *Arch Gynecol Obstet* 2016;294:945-52.

42. Chou D, Abalos E, Gyte GM, et al. Drugs for perineal pain in the early postpartum period

For peer review only

For peer review only - http://bmjopen.bmj.com/site/about/guidelines.xhtml

(Protocol). *Cochrane Database Syst Rev* 2016;3: CD007734.

43. Persico G, Vergani P, Cestaro C, et al. Assessment of postpartum perineal pain after vaginal delivery: prevalence, severity and determinants. A prospective observational study. *Minerva Ginecol* 2013;65:669-78.

44. Safadi RR, Abushaikha LA, Ahmad MM. Demographic, maternal, and infant health correlates of post-partum depression in Jordan. *Nurs Health Sci* 2016;18:306-13.

45. Cooklin AR, Amir LH and Nguyen CD, et al. Physical health, breastfeeding problems and maternal mood in the early postpartum: a prospective cohort study. *Arch Womens Ment Health* 2018;21:365-374.

46. Fodstad K, Staff AC, Laine K. Sexual activity and dyspareunia the first year postpartum in relation to degree of perineal trauma. *Int Urogynecol J* 2016;27:1513-23.

47. Klein MC, Gauthier RJ, Robbins JM, et al. Relationship of episiotomy to perineal trauma and morbidity, sexual dysfunction, and pelvic floor relaxation. *Am J Obstet Gynecol* 1994;171:591-8.

48. Salmon D. A feminist analysis of women's experiences of perineal trauma in the immediate post-delivery period. *Midwifery* 1999;15:247-56.

49. İlhan G, Verit Atmaca FF, Eken M, et al. What is Turkish women's opinion about vaginal delivery? *Turk J Obstet Gynecol* 2015;12:75-8.

50. Serçekuş P and Okumuş H.Fears associated with childbirth among nulliparous women in Turkey. *Midwifery* 2009;25:155-162.

51. Crookall R, Fowler G, Wood C, et al. A systematic mixed studies review of women's experiences of perineal trauma sustained during childbirth. *J Adv Nurs* 2018 May 23. DOI: 10.1111/jan.13724

52. Priddis H, Schmied V, Dahlen H. Women's experiences following severe perineal trauma: a qualitative study. *BMC Womens Health* 2014;14:32.

53. Williams A, Lavender T, Richmond DH, et al. Women's experiences after a third-degree obstetric anal sphincter tear: a qualitative study. *Birth* 2005;32:129-36.

For peer review only

For peer review only - http://bmjopen.bmj.com/site/about/guidelines.xhtml

Reporting checklist for qualitative study.

Based on the SRQR guidelines.

Instructions to authors

Complete this checklist by entering the page numbers from your manuscript where readers will find each of the items listed below.

Your article may not currently address all the items on the checklist. Please modify your text to include the missing information. If you are certain that an item does not apply, please write "n/a" and provide a short explanation.

Upload your completed checklist as an extra file when you submit to a journal.

In your methods section, say that you used the SRQRreporting guidelines, and cite them as:

O'Brien BC, Harris IB, Beckman TJ, Reed DA, Cook DA. Standards for reporting qualitative research: a synthesis of recommendations. Acad Med. 2014;89(9):1245-1251.

		Reporting Item	Page Number
**Title**			
	#1	Concise description of the nature and topic of the study identifying the study as qualitative or indicating the approach (e.g. ethnography, grounded theory) or data collection methods (e.g. interview, focus group) is recommended	1
**Abstract**			
	#2	Summary of the key elements of the study using the abstract format of the intended publication; typically includes background, purpose, methods, results and conclusions	3
**Introduction**			
Problem formulation	#3	Description and significance of the problem / phenomenon studied: review of relevant theory and empirical work; problem statement	5

For peer review only

Purpose or research question	#4	Purpose of the study and specific objectives or questions	6
**Methods**			
Qualitative approach and research paradigm	#5	Qualitative approach (e.g. ethnography, grounded theory, case study, phenomenolgy, narrative research) and guiding theory if appropriate; identifying the research paradigm (e.g. postpositivist, constructivist / interpretivist) is also recommended; rationale. The rationale should briefly discuss the justification for choosing that theory, approach, method or technique rather than other options available; the assumptions and limitations implicit in those choices and how those choices influence study conclusions and transferability. As appropriate the rationale for several items might be discussed together.	6,7
Researcher characteristics and reflexivity	#6	Researchers' characteristics that may influence the research, including personal attributes, qualifications / experience, relationship with participants, assumptions and / or presuppositions; potential or actual interaction between researchers' characteristics and the research questions, approach, methods, results and / or transferability	11
Context	#7	Setting / site and salient contextual factors; rationale	7
Sampling strategy	#8	How and why research participants, documents, or events were selected; criteria for deciding when no further sampling was necessary (e.g. sampling saturation); rationale	7,8
Ethical issues pertaining to human subjects	#9	Documentation of approval by an appropriate ethics review board and participant consent, or explanation for lack thereof; other confidentiality and data security issues	8
Data collection methods	#10	Types of data collected; details of data collection procedures including (as appropriate) start and stop dates of data collection and analysis, iterative	8,9

For peer review only

		process, triangulation of sources / methods, and modification of procedures in response to evolving study findings; rationale	
Data collection instruments and technologies	#11	Description of instruments (e.g. interview guides, questionnaires) and devices (e.g. audio recorders) used for data collection; if / how the instruments(s) changed over the course of the study	8,9
Units of study	#12	Number and relevant characteristics of participants, documents, or events included in the study; level of participation (could be reported in results)	7
Data processing	#13	Methods for processing data prior to and during analysis, including transcription, data entry, data management and security, verification of data integrity, data coding, and anonymisation / deidentification of excerpts	10
Data analysis	#14	Process by which inferences, themes, etc. were identified and developed, including the researchers involved in data analysis; usually references a specific paradigm or approach; rationale	10
Techniques to enhance trustworthiness	#15	Techniques to enhance trustworthiness and credibility of data analysis (e.g. member checking, audit trail, triangulation); rationale	10
**Results/findings**			
Syntheses and interpretation	#16	Main findings (e.g. interpretations, inferences, and themes); might include development of a theory or model, or integration with prior research or theory	13-20
Links to empirical data	#17	Evidence (e.g. quotes, field notes, text excerpts, photographs) to substantiate analytic findings	21-25
**Discussion**			
Intergration with prior work, implications, transferability and contribution(s) to the field	#18	Short summary of main findings; explanation of how findings and conclusions connect to, support, elaborate on, or challenge conclusions of earlier scholarship; discussion of scope of application /	25-28

For peer review only

		generalizability; identification of unique contributions(s) to scholarship in a discipline or field	
Limitations	#19	Trustworthiness and limitations of findings	25,26
**Other**			
Conflicts of interest	#20	Potential sources of influence of perceived influence on study conduct and conclusions; how these were managed	29
Funding	#21	Sources of funding and other support; role of funders in data collection, interpretation and reporting	29

The SRQR checklist is distributed with permission of Wolters Kluwer © 2014 by the Association of American Medical Colleges. This checklist was completed on 31. July 2019 using https://www.goodreports.org/, a tool made by the EQUATOR Network in collaboration with Penelope.ai

For peer review only

Characteristics of health care providers

	Health care providers
**Age (year)**	
Mean±SD	40.8±5.0
Range	29~52
**Occupation**	
Obstetrician	4
Midwife	5
Community health care provider	3
**Obstetric Institution**	
Municipal MCH Hospital	2
Tertiary general hospital	2
District MCH Hospital	3
Secondary general hospital	3
Community health center	2
**Work experience (year)**	
Mean±SD	16.8±8.2
Range	3~28

For peer review only

Themes and illustrative quotes

Themes	Quotes
**Women's knowledge of the procedure varies considerably**	"I barely knew anything about episiotomy before the delivery."(#6, 28 years old, primipara, six months after childbirth)<br>"The doctors didn't inform me about the procedure(episiotomy). After childbirth, the woman in the same delivery ward asked me 'did you get episiotomy' and I reply 'what's the episiotomy?' I didn't know it before and I finally realized what the anesthesia and suturing meant at that time." (#9, 33 years old, primipara, four days after childbirth)<br>"I was ready to have a normal vaginal birth···When the day came, the baby was stuck because the perineal skin was very tight. [Therefore, I had an episiotomy.] I used to wonder what episiotomy is, and only came to know exactly what it is after childbirth. I basically had no idea about episiotomy before childbirth, and at that time I know it -- Oh, this is episiotomy!" (#14, 28 years old, primipara, one week after childbirth)<br>"I think it is necessary to do episiotomy when it can accelerate the progress of labor. But if the baby can be delivered smoothly, episiotomy should be avoided. After all, it is still a surgery." (#11, 30 years old, primipara, two months after childbirth)<br>"The hospital takes episiotomy as a routine practice during normal vaginal birth. I think if episiotomy can relieve your suffering, routine episiotomy should be recommended. I felt that my perineum recovered soon after episiotomy. On the other hand, episiotomy won't cause any big problems, as long as you move carefully and clean yourself frequently." (#26, 28 years old, primipara, two weeks after childbirth)<br>"The doctor said that my uterine contractions were too weak, but I didn't feel that way. I just needed some time. I don't like the episiotomy at all. I searched episiotomy on the Internet and found its rate in China is excessively high. Many situations are not necessary. The doctors might be afraid of potential risks. I think if there is nothing wrong with the puerpera, the episiotomy should be avoided as far as possible. Previous generation like my mother didn't use episiotomy but they recovered quite well." (#8, 34 years old, primipara, two months after childbirth)
**The pain interferes with daily life for weeks**	"I still feel pain of my perineal wound now and I can feel the difference between the two sides of perineum···the right side with the episiotomy lack skin elasticity. And there was an obvious scar in my perineum when I had the postpartum check-up at 42 days after childbirth." (#1, 35 years old, multipara, EP with forceps, six months after childbirth)

For peer review only - http://bmjopen.bmj.com/site/about/guidelines.xhtml

	" When I tried to sit down, it seems like that I was sitting on a knife. The wound seemed to split. It hurt that much." (#16, 32 years old, primipara, two years after childbirth)<br><br>"The wound hurt in the first few days. Five days after delivery, I started to feel better, but I can still feel the pulling or tugging pain at the incision···It was a bit tight." (#26, 28 years old, primipara, two weeks after childbirth)<br><br>"I had to move slowly because I felt painful when I moved suddenly. （#12, 40 years old, multipara, two months after childbirth）<br><br>"The doctor said I must sleep on the side. It was uncomfortable to sleep one-sided for a long time." (#6, 28 years old, primipara, six months after delivery)<br><br>"The healing was not very good [of my perineum], because the incision was relatively big. I went to emergency room twice within the first month [after childbirth]. The reason was the wound got infected. After removing the stiches, I was getting recovered from the cut. In the first few days, I was fed by my mother. I couldn't sit, and I just lay down there. I ate on the bed in the first month. " (#20, 30 years old, primipara, EP with forceps delivery, two months after childbirth)<br><br>"My cut was six centimeters and it was done by a young midwife. The wound split at the six day after birth, then I suffered a lot because it recovered slowly. The pain had continued for half a month and the stiches cannot be absorbed ···At that time, I couldn't sit or squat, and I had to move very slowly. Even now, I am still feeling painful when I am sitting" (#8, 34 years old, two months after childbirth)<br><br>"My wound hurt very much in the first week, and I couldn't peep or poop at all because I couldn't sit on the toilet. Every time using the toilet was like a torture to me. I think that most women who have received an episiotomy would probably have the same problem as me." (#1, 35 years old, multipara, EP with forceps, six months after childbirth)<br><br>I didn't want to use the toilet for bowel movement [defecation] until I had to poop···the fourth day after childbirth···The wound was so painful and I feared it would split when I exerted to poop···I also got hemorrhoids at that time, which became an obstacle of bowel movement. (#26, 28 years old, primipara, two weeks after childbirth)<br><br>"I just felt the wound painful during breastfeeding. Sometimes I felt that my baby couldn't get the nipples, so I just leaned forward a bit, and suddenly, the episiotomy wound began to hurt. After all, there were two cuts in my perineum [The women also got episiotomy at her last childbirth] ···and I don't like to lie down [to feed the baby]." (#4, 34 years

For peer review only

	old, multipara, two months after childbirth)<br><br>"It was very tiring and painful to sit down··· I felt my wound was also swollen, and I had to sit on one-side, lean my body to the side without episiotomy. I sat in this way for the breastfeeding within the whole first month ···. This made my back hurt and sometimes it was really awful."<br><br>(#28, 21 years old, primipara, six months after childbirth)<br><br>"···. If the wound gets infected because of improperly sterilization during the procedure, it would be very troublesome. The healing will take one to three weeks. In this kind of case, women with episiotomy would be more tortured than those with C-section." (Obstetrician, 28 years of relevant work experience, district MCH hospital.)<br><br>"Catgut (a kind of stitches) absorption varies from people to people, and some women may be allergic··· have catgut rejection. If the sutures cannot be absorbed, it is always a foreign matter in perineum, which will hurt. " (Midwife, 25 years of relevant work experience, district MCH hospital)<br><br>"Episiotomy does have impacts on daily activities, such as breastfeeding. Some women are unwilling to breastfeed while lying down, or they just don't know how to breastfeed while lying down. Sometimes, people would feel anxious because of the pain. The milk secretion could also be affected by the pain." (Midwife, 20 years of relevant work experience, secondary general hospital)<br><br>"Walking and breastfeeding could be affected [by EP]. Episiotomy has a major impact on breastfeeding, because some women have short nipples, and it is difficult for the baby to suck the nipples when in lying position. Thus, it is easier to breastfeed with sitting position (Community health care provider, 17 years of relevant work experience, community health center)
**Long term anxiety is a consequence for some, described as a "psychological shadow"**	"We used the method of withdraw when having sex, because the inside of my perineum was uneven and rough, which hooked the condom and affected intercourse." (#13, 39 years old, primipara, seven years after childbirth)<br><br>"We returned to sexual life quite late, about half a year after delivery. The first three or four times [of sexual life] turned out painful because of the wound and vaginal dryness. When my baby was 10 months old, it wasn't painful anymore." (#23, 29 years old, primipara, 14 months after childbirth)<br><br>"Because of the terribly perineal pain, I asked my husband to resume sexual life a year later. I didn't dare to do it, because I worried the wound

For peer review only - http://bmjopen.bmj.com/site/about/guidelines.xhtml

	would pain again." (#16, 32 years old, primipara, two years after childbirth)<br><br>"Psychologically, I feel that the vagina cannot recover to original status···you feel the vagina is looser than before. And your spouse also has some psychological barriers to postpartum sexual life. I feel that many mothers who undergo episiotomy will have the shadows of sexual life more or less. The psychological shadow might disappear over time, but I don't know yet. (#1, 35 years old, multipara, six months after childbirth)<br><br>"The biggest problem for me now is the next delivery. Will I receive an episiotomy again? Will my second vaginal delivery encounter difficulties because of my rough perineum [the wound of episiotomy this time]?" (#3, 32 years old, primipara, EP with forceps, 14 months after childbirth)<br><br>" I don't dare to deliver my second child through normal birth (vaginal delivery). The experience of recovering from the episiotomy was indeed miserable. It really scared me. Maybe not having a second child is better··· or maybe I would choose C-section even though it has some negative effects. ······ if I had a vaginal birth again, and an episiotomy again. I cannot imagine what will happen, my vagina would be totally 'useless' for sexual life." (#13, 39 years old, primipara, two years after childbirth)<br><br>"The doctor directly did the episiotomy at my first childbirth. So I gained some childbirth experience and I was always afraid that I would suffer episiotomy again during this childbirth. There was a psychological shadow when I thought of the childbirth··· I was worried about these problems such as deliver again, episiotomy again, miserable recovery of episiotomy. Finally, I still got episiotomy again!" (#29, 30 years old, multipara, six months after childbirth)<br><br>"Some women can't have intercourse at all. Once I met a case that the vagina of the women so tight that I can' even put one finger in, due to the scar contracture." (Obstetrician,28 years of relevant work experience, district MCH hospital)<br><br>"It doesn't have a lot of affection, because interval between births is generally long. It takes at least one year, right? The skin would recover within a year.···it's just that the wound will look ugly but the birth process won't be affected." (Midwife, 25 years of relevant work experience, district MCH hospital)<br><br>"Some people are scar physique (a kind of people who easily have enormous scar). This kind of scar is hard and protuberant so that we fear the wound would tear again during the second childbirth. If the scar tear

For peer review only - http://bmjopen.bmj.com/site/about/guidelines.xhtml

	and was sew up again, it can't heal very well. (Midwife, 25 years of work experience, secondary general hospital)<br><br>"What are the impacts of episiotomy on further childbirth? It is true that it may cast a psychological shadow on those women. If the episiotomy wound from first childbirth is infected or she had a severe tear, she won't dare to have another child, or she might choose C-section. Next time she might say: 'I don't want another baby' and 'I want to choose C-section instead of vaginal delivery."(Obstetrician, 28 years of relevant work experience, district MCH hospital)
**Societal norms assume women will not complain**	"Whenever I said I felt sore of the perineal wound, they would say, 'why you still feel painful after 4 months?'. It sounds like I shouldn't be sore. Every time my husband said these words, I would response to him, 'you should get a cut and experience the healing process." (#28, 21 years old, primipara, six months after childbirth)<br><br>"I wondered if all the women would have the perineal pain after the childbirth···They [family members], such as my sister in law said that I was a bit low-tolerant, ...they all had birth experience but they never heard that a puerpera unable to sit down after childbirth. At that time, I felt it was so hard to be a woman." (#16, 32 years old, primipara, two years after childbirth)<br><br>"No, I'm not very low-tolerant. Even if it hurts, I would endure the pain and not mention it. It didn't hurt that much. I could still bear with it. Some women are too spoiled to bear any pain and they always groan, which I thought it is meaningless. Nobody could replace your sufferings. It's normal thing, also the destiny of every woman." (#29, 30 years old, multipara, six months after childbirth)<br><br>"It was painful during suturing. It was the sharp tingling when I got the cut but the pain of the childbirth was more painful than this. After local anesthetic, it was still very painful and he sewed a few stitches. But I was immersed in the joy of having a baby at that time so I felt the pain could be tolerated···but I was trembling because of pain and the doctor comforted me 'it would be finished soon, you can bear it, yes?" (#23, 29 years old, primipara, 14 months after delivery)<br><br>"I felt no pain about the cut because of the anesthetic···the suturing process was more painful. I cannot keep unmoved because the anesthetic effects tailed off later. And the doctor kept telling me not to move, saying that he couldn't sew up well if I still move. But it was painful and he was sewing up for a long time because my wound was very big...I couldn't stay still, and I didn't know whether the stitches were done properly. I don't know if it related to my unabsorbed suturing knot,

For peer review only - http://bmjopen.bmj.com/site/about/guidelines.xhtml

	maybe it resulted from my own body condition (some immune factor). Doctors also can't change anything(sigh)" (#20, 30 years old, primipara, EP with forceps delivery, two months after delivery)<br><br>"I felt painful so much! I thought I really needed some treatments to relive the pain but the doctor thought I could endure this kind of pain……I really can't endure it since my wound is very large. I hadn't fallen asleep for several days after childbirth. The pain was so awful!" (#20, 30 years old, primipara, EP with forceps, two months after childbirth)

For peer review only

For peer review only - http://bmjopen.bmj.com/site/about/guidelines.xhtml

BMJ Open

Women's experience of episiotomy: a qualitative study from China

Journal:	*BMJ Open*
Manuscript ID	bmjopen-2019-033354.R2
Article Type:	Original research
Date Submitted by the Author:	03-Apr-2020
Complete List of Authors:	He, Siyuan; Fudan University, Maternal, Child and Adolescent Health, School of Public Health; Shanghai Municipal Center for Health Promotion<br>Jiang, Hong; Fudan University, Maternal, Child and Adolescent Health, School of Public Health<br>Qian, Xu; Fudan University, Maternal, Child and Adolescent Health, School of Public Health<br>Garner, Paul; Liverpool School of Tropical Medicine, Centre for Evidence Synthesis in Global Health, Department of Clinical Sciences
**Primary Subject Heading**:	Obstetrics and gynaecology
Secondary Subject Heading:	Sexual health, Qualitative research, Public health
Keywords:	QUALITATIVE RESEARCH, PRIMARY CARE, Community gynaecology < GYNAECOLOGY, GYNAECOLOGY

SCHOLARONE™
Manuscripts

I, the Submitting Author has the right to grant and does grant on behalf of all authors of the Work (as defined in the below author licence), an exclusive licence and/or a non-exclusive licence for contributions from authors who are: i) UK Crown employees; ii) where BMJ has agreed a CC-BY licence shall apply, and/or iii) in accordance with the terms applicable for US Federal Government officers or employees acting as part of their official duties; on a worldwide, perpetual, irrevocable, royalty-free basis to BMJ Publishing Group Ltd ("BMJ") its licensees and where the relevant Journal is co-owned by BMJ to the co-owners of the Journal, to publish the Work in this journal and any other BMJ products and to exploit all rights, as set out in our licence.

The Submitting Author accepts and understands that any supply made under these terms is made by BMJ to the Submitting Author unless you are acting as an employee on behalf of your employer or a postgraduate student of an affiliated institution which is paying any applicable article publishing charge ("APC") for Open Access articles. Where the Submitting Author wishes to make the Work available on an Open Access basis (and intends to pay the relevant APC), the terms of reuse of such Open Access shall be governed by a Creative Commons licence – details of these licences and which Creative Commons licence will apply to this Work are set out in our licence referred to above.

Other than as permitted in any relevant BMJ Author's Self Archiving Policies, I confirm this Work has not been accepted for publication elsewhere, is not being considered for publication elsewhere and does not duplicate material already published. I confirm all authors consent to publication of this Work and authorise the granting of this licence.

For peer review only

Title page

Title: Women's experience of episiotomy: a qualitative study from China

*Corresponding author: Xu Qian, Mailbox 175, 138 Yixueyuan Road, Shanghai, 200032, China; email: xqian@shmu.edu.cn; Tel: +86 021 5427267

All co-authors:

#Siyuan He

Department of Maternal, Child and Adolescent Health, School of Public Health, Fudan University, Shanghai, China.

Shanghai Municipal Center for Health Promotion, Shanghai, China.

#Hong Jiang

Department of Maternal, Child and Adolescent Health, School of Public Health, Fudan University, Shanghai, China.

*Xu Qian

Department of Maternal, Child and Adolescent Health, School of Public Health, Fudan

For peer review only

For peer review only - http://bmjopen.bmj.com/site/about/guidelines.xhtml

University, Shanghai, China.

Paul Garner

Centre for Evidence Synthesis in Global Health, Department of Clinical Sciences,

Liverpool School of Tropical Medicine, Liverpool, UK.

SH and HJ contributed equally to this work.

Word count：6111 words

For peer review only

For peer review only - http://bmjopen.bmj.com/site/about/guidelines.xhtml

ABSTRACT

Objectives To describe women's experience of episiotomy in urban China.

Design Semi-structured, in-depth interviews with women after episiotomy. We analyzed transcriptions using thematic analysis in Chinese. Emerging themes were debated in English to finalize interpretation.

Settings Two community health centers and four hospitals in Shanghai, China.

Participants Purposive sampling of 30 postpartum women who had experienced episiotomy; twenty-five were primiparous, and four had deliveries by forceps. We interviewed health providers to complement the data.

Results We identified four main themes: a) Women's views of the procedure vary considerably; b) The pain interferes with daily life for weeks; c) Long term anxiety is a consequence for some, described as a "psychological shadow"; d) Societal norms assume women will not complain.

Conclusion Women receive little information in advance about episiotomy, yet the procedure has a wide range of physical and psychological consequences. This includes long term anxiety about the damage done to them as women.

Key words: episiotomy, perineal trauma, women's experience

Strengths and Limitations of this study

For peer review only

For peer review only - http://bmjopen.bmj.com/site/about/guidelines.xhtml

- This study was one of a few qualitative studies to explore women’s experience of episiotomy after childbirth
- The study identified an effect of episiotomy described in Chinese as a "psychological shadow”, and that societal norms meant women felt they were expected to suffer alone and not complain
- We interviewed women at different times after episiotomy, and were not able to evaluate whether their perceptions changed over time

For peer review only - http://bmjopen.bmj.com/site/about/guidelines.xhtml

INTRODUCTION

Doctors introduced episiotomy as a surgical procedure in the 1950's to reduce the risk of severe perineal tear, shorten delivery, and prevent damage to the pelvic floor.[1] However, the procedure can cause pain in the immediate postpartum period, the wound can become infected, and the scar can cause long-term dyspareunia. Indeed, the benefits of routine episiotomy have been contested.[2] This balance between benefits and harms has been evaluated in randomized controlled trials. These are summarized in the Cochrane review, and this shows that there is no evidence that routine episiotomy has the benefits originally assumed; and that more restricted use results in fewer women experiencing severe perineal or vaginal trauma.[3]

International institutions and professional societies now recommend episiotomy only when there is a clear clinical indication.[4-7] Practices in most European countries[8] show rates have fallen. However, episiotomy rates in vaginal births are still high in some countries; for example, 53.2% in Chile[9] (from hospital records), 73% from a hospital in Lebanon,[10] and 92% from a hospital in Cambodia.[11]

Pushback from consumers in the early 1990's may have contributed to the decline in routine episiotomy in the UK, but in general recommendations for episiotomy have been set up mainly from the health provider's medical standpoint, with little reference to the views or preferences of women. The Cochrane review (2017) pointed out that trials inadequately considered women's preferences, views on the procedures, or the outcomes that are important to them.[3]

For peer review only - http://bmjopen.bmj.com/site/about/guidelines.xhtml

More recently, the World Health Organization recognized the need for a “positive childbirth experience”,[4] which corresponds to the new Global Strategy for Women’s, Children’s and Adolescents’ Health (2016–2030). With an increase in emphasis to women-centered outcomes in clinical decision making, women’s experience of episiotomy is highly relevant.

In China, episiotomy used to be a routine practice for vaginal deliveriy.[12 13] In the last decade, hospital data reported levels of 47.4% to 84.7%;[14-17] and, some multi-center studies reported hospital rates from 41.2% to 69.7%.[18 19] For China, where there were 17.23 million births in 2016,[20] there could be as many as 7.33 million episiotomies a year (given a vaginal birth rate of 61.0% and episiotomy rate of 69.7% among vaginal birth).[18] Although the Chinese national obstetric guideline has recommended restrictive use of episiotomy since 2016, it has not been implemented.[21]

We found no data in the published English literature on the experience of episiotomy in women in China, and therefore carried out this study. Through this qualitative study, we aimed to describe how women experience in urban China.

METHODS

Approach, setting and sampling strategy

We used standard qualitative methods with semi-structured, in-depth individual interviews. The details of the methods were reported according to the SRQR reporting

For peer review only

For peer review only - http://bmjopen.bmj.com/site/about/guidelines.xhtml

checklist (see Supplementary File 1).[22] We conducted the study in Shanghai (population 24.2 million (2016)). The city has had a policy of routine episiotomy from 1999; and the rate around 35.8% to 86.67% from 2011 to 2014.[23-25] Community health centers hold pregnancy registration and information to allow home visits in the postnatal period for clients in their catchment area, while clinical services for childbirth are provided by higher level of hospitals, and episiotomy practices vary between hospitals. Compared with general hospitals, maternal and child health (MCH) hospitals are more likely to adopt restrictive episiotomy policy since their midwives are experienced and well trained, and women at these settings tend to be low-risk. We used the two community health centers where we had worked previously and thus staff were familiar with us: one in Pudong District, east of Shanghai (1459 pregnant women registered in 2017); and one in Xuhui District, west of Shanghai (775 pregnant women registered in 2017).

We used purposive sampling strategy, seeking women over 18 years old who had undergone an episiotomy in her last birth. We recruited women from three different postpartum periods (within two weeks, no more than six months and six months above after childbirth); we also took account of the types of hospitals to ensure a mix of experiences. Women being invited for this study delivered in various types of hospitals including municipal MCH hospital, tertiary general hospital, district MCH hospital and secondary general hospital. Experienced health care providers who had over three years work experience in maternal health area were recruited to confirm women's symptoms and help to better understand women's views and reflections. Two or three health care

For peer review only - http://bmjopen.bmj.com/site/about/guidelines.xhtml

providers from each type of hospitals were involved in this study and their characteristics are shown in the Supplementary File 2. We stopped interviewing women when we appeared not to identify new information.[26-28] The primary researcher (SH) carried out the interviews, under the guidance of the supervisors (HJ and XQ). SH is a master student, who had received training in qualitative methods and had a six-month work placement with the MCH administration.

Ethical approval

All participants were informed about the research purpose and contents. Interviews were conducted after written informed consent obtained from each participant. The research obtained the approval from the Institutional Review Board in School of Public Health, Fudan University.

Patient and public involvement

When designing the study, we invited a few women to give us feedback on the approach and the questions to ask. We collected women's comments on the public internet forum and interviewed four women about the research topic before we designed the interview guide. This preliminary work led to several revisions to the interview guide.

Data collection

We approached women by accompanying health staffs during postpartum home visits or when women brought their children for child health checkup in community health centers

For peer review only

For peer review only - http://bmjopen.bmj.com/site/about/guidelines.xhtml

between September 2017 and March 2018. We used an interview guide based on the literatures and our research group discussions (Table 1);[29-31] also, we reviewed women's comments on the public internet forum to improve the design of interview guide and piloted the interview guide with four postpartum women. The piloted data were also included in our analysis as it was consistent with the main sample. Interviews were conducted in private rooms in the community health centers, hospitals and interviewees' homes and all women provided signed consents. Interviews were in Chinese and recorded with permission. For the health providers, they were recommended by relevant administrators and invited to this study. They were interviewed at a private room in their workplaces.

Table 1 Interview guide

What's your experience after episiotomy, from the childbirth to postpartum period? (Probe: discomfort, pain, swelling)
Did episiotomy impact on your daily life? How? (Probe: walking, sitting, breastfeeding, baby care, sexual life, medication, mood)
How did you deal with your suffering or problems? (Probe: medical services usage)
Are there some long-lasting effects of episiotomy you have noticed? If yes, what are they?

Data analysis

For peer review only - http://bmjopen.bmj.com/site/about/guidelines.xhtml

Medical master students transcribed interviews, and one of the interviewers (SH) checked them for accuracy. We used NVivo8.0 (QSR) software for thematic analysis.[32 33] Two researchers (SH and YC) read all the transcripts and coded the data to identify the reoccurring topics, ideas, or concepts independently. After discussing the differences of the coding, they organized the data into initial themes. Initial themes and quotes were translated into English and checked by XQ. All the co-authors then further reflected on these themes and developed overarching categories, discussing the themes in both Chinese and English. The health professionals' responses were grouped against the emergent themes from the women's interviews and included within corresponding themes.

During this process, on two occasions we found themes that we could not translate directly into English. Rather than being a problem, these were both informative and underlying themes. The team discussed the words carefully in Chinese and English to gain a common understanding of meaning and cultural context.

The research team included three bilingual speakers (SH, HJ, XQ) and one native English speaker (PG). All the themes, descriptions and corresponding quotes were checked by all the authors.

Reflexivity

As a team, we discussed our prior beliefs and experiences in early discussions and during analysis to reflect on how this may influence our analysis. The research team

For peer review only - http://bmjopen.bmj.com/site/about/guidelines.xhtml

included people that had performed, repaired and experienced episiotomy (HJ, QX, PG). Evaluating episiotomy and the uncertainty around benefits and harms is a topic of interest to all the authors, and, as with many medical and obstetrical interventions, we as researchers remain “healthy sceptics”. Three authors have completed the Cochrane review examining this topic (HJ, QX, PG) and reporting is that consumer views on the procedure are important for medical policy. All had experience in collecting and analyzing qualitative data; PG and QX have worked together for over 20 years on projects about whether obstetric practice and research evidence are in alignment in China.

RESULTS

We interviewed 30 postpartum women, age range from 21 to 40 (mean age 30.1) years. Twenty-five women were primiparous; all had experienced episiotomy and four also received assisted delivery with forceps. Seven women were interviewed within two weeks, nine were at two months, and fourteen were above six months after childbirth (Table 2). Four main themes emerged: a) Women’s views of the procedure vary considerably; b) The pain interferes with daily life for weeks; c) Long term anxiety is a consequence for some, described as a “psychological shadow”; d) Societal norms assume women will not complain. The complete illustrative quotes were shown in the Supplementary File 3.

Table 2 Characteristics of the Postpartum Women

	Women

For peer review only - http://bmjopen.bmj.com/site/about/guidelines.xhtml

**Age (year)**	
Mean±SD	30.1±3.8
Range	21~40
**Parity**	
Primipara	25
Multipara	5
**Mode of delivery**	
Episiotomy	26
Episiotomy with forceps	4
**Interview time**	
Within two weeks after childbirth	7
No more than six months after childbirth	9
Six months above after childbirth	14

1. Women's views of the procedure vary considerably

This theme describes women's various views of episiotomy including their knowledge, feelings, and altitudes. The theme also explains how women's views are influenced from childbirth to postpartum period.

1.1 "What is episiotomy?"

In general, women had little knowledge about episiotomy before childbirth, indicating that they were not well informed. Inadequate knowledge made women under a kind of fear

For peer review only - http://bmjopen.bmj.com/site/about/guidelines.xhtml

before childbirth, while sound knowledge capacitated women to realize both the benefits and harms so that they considered episiotomy more justly. Nearly one fifth of women knew very little about the procedure before childbirth. For some, their understanding of episiotomy was attained through their laboring experiences and the people around them. They even did not know what happened until other people told them, or just heard the name of this obstetric intervention.

“The doctors didn’t inform me about the procedure (episiotomy). After childbirth, the woman in the same delivery ward asked me ‘did you get episiotomy’ and I reply ‘what’s the episiotomy?’ I didn’t know it before and I finally realized what the anesthesia and suturing meant at that time.” (#9, 33 years old, primipara, four days after childbirth)

"... I used to wonder what episiotomy is, and only came to know exactly what it is after childbirth… at that time [When I was cut] I know it -- Oh, this is episiotomy!"   (#14, 28 years old, primipara, one week after childbirth)

"At that time, I thought, 'Oh my god! They will certainly cut my vulva. The vulva would be ugly and [its function would be] affected!' It sounds scary." (#28, 21 years old, primipara, six months after childbirth)

A few women seemed to be more informed, from a variety of sources: online resources, discussion with other women, and from doctors. These more informed women were able to express the concept of balancing benefits and harms in their conversations:

For peer review only - http://bmjopen.bmj.com/site/about/guidelines.xhtml

"I think it is necessary to do episiotomy when it can accelerate the progress of labor. But if the baby can be delivered smoothly, episiotomy should be avoided. After all, it is still a surgery." (#11, 30 years old, primipara, two months after childbirth)

1.2 Two contrasting altitudes towards the policy of episiotomy

There were opposite opinions about the policy of episiotomy. Women's personal recovery experience was, unsurprisingly, significant in shaping their views: some clearly supported routine episiotomy, while others criticized this as an excessive obstetric intervention. One woman accepted routine episiotomy was required, and another multipara who had an episiotomy with her first childbirth requested it for her second delivery. These women had few problems with their current procedures appeared to accept the need for the procedure. However, those who had a miserable experience seemed more likely to complain the negative effects and question the need for an episiotomy. Two quotes below typically represent these two situations:

"The hospital takes episiotomy as a routine practice during normal vaginal birth. I think if episiotomy can relieve your suffering, routine episiotomy should be recommended. I felt that my perineum recovered soon after episiotomy. On the other hand, episiotomy won't cause any big problems, as long as you move carefully and clean yourself frequently." (#26, 28 years old, primipara, two weeks after childbirth)

"The doctor said that my uterine contractions were too weak, but I didn't feel that way. I just needed some time. I don't like the episiotomy at all. I searched episiotomy on

For peer review only - http://bmjopen.bmj.com/site/about/guidelines.xhtml

the Internet and found its rate in China is excessively high. Many situations are not necessary. The doctors might be afraid of potential risks." (#8, 34 years old, primipara, two months after childbirth)

2. The pain interferes with daily life for weeks

This theme describes women's pain after episiotomy and how it influences their postpartum daily life widely.

2.1 Pain from episiotomy varied

Women's pain and discomfort varied-but in some was severe, and in a few lasted for months. Women in pain for two weeks only described the pain as "a little pain or discomfort", but a few women reported considerable pain for months after childbirth, three reporting this as "intolerable" for more than one month. These women with severe pain also reported problems with suturing including tight stitches, irritation from the stitches or the wound gaping.

"I still feel pain of my perineal wound now and I can feel the difference between the two sides of perineum... the right side with the episiotomy lack skin elasticity... " (#1, 35 years old, multipara, EP with forceps, six months after childbirth)

"The wound hurt in the first few days. Five days after delivery, I started to feel better, but I can still feel the pulling or tugging pain at the incision... it was a bit tight." (#26, 28 years old, primipara, two weeks after childbirth)

"The wound split at the six day after birth, then I suffered a lot because it recovered

For peer review only - http://bmjopen.bmj.com/site/about/guidelines.xhtml

For peer review only

slowly. The pain had continued for half a month and the stiches cannot be absorbed... Even now, I am still feeling painful when I am sitting" (#8, 34 years old, two months after childbirth)

2.2 Restricted postures and movements

Avoiding pain, and fear that the episiotomy would split, meant women avoided moving around. Women stated they were conscious of the wound and avoiding pain, so had to walk or move slowly, or avoid contact as the wound hurt when pressed. Some had to sit or lie on one-side or stay in one position for a long time to avoid pain, and this made them tired and uncomfortable. Three women with problems with the episiotomy healing complained that they could not sit down for a minute because of the horrible pain, which greatly influenced their postpartum life such as sleeping and eating.

"At that time (half a month after childbirth), I couldn't sit or squat [because of the horrible pain], and I had to move very slowly." (#8, 34 years old, two months after childbirth)

"The healing was not very good [of my perineum] ... in the first few days, I was fed by my mother. I couldn't sit [because of pain], and I just lay down there. I ate on the bed in the first month. " (#20, 30 years old, primipara, EP with forceps delivery, two months after childbirth)

2.3 Obvious difficulties of breastfeeding and defecation

The pain of episiotomy brought various life impacts to women. Among these,

For peer review only - http://bmjopen.bmj.com/site/about/guidelines.xhtml

breastfeeding and defecation were mentioned a lot. Several volunteered pain from episiotomy interfered breastfeeding. Usually, women liked to feed baby whilst sitting, if this was painful then they struggled to feed. Some of them learned to breastfeed by lying down or using breast pump in a standing position. Other women sat in pain and found it a struggle, increasing the difficulty and fatigue of breastfeeding. The pain often interfered with defecation, with increasing pain and the sensation of the wound about to split whilst defecating. Just sitting or squatting was already hard. This fear of pain or that the wound would split open led women to avoid defecation, worsening existing postpartum constipation.

> *"It was very tiring and painful to sit down... I felt my wound was also swollen, and I had to sit on one-side, lean my body to the side without episiotomy. I sat in this way for the breastfeeding within the whole first month... this made my back hurt and sometimes it was really awful." (#28, 21 years old, primipara, six months after childbirth)*

> *"My wound hurt very much in the first week, and I couldn't peep or poop at all because I couldn't sit on the toilet (This posture the pulls the wound). Every time using the toilet was like a torture to me. I think that most women who have received an episiotomy would probably have the same problem as me." (#1, 35 years old, multipara, EP with forceps, six months after childbirth)*

Health providers had different views on the women's experience. Meanwhile, they partly explained the reason for long-term perineal pain and how it affected physical functions.

For peer review only - http://bmjopen.bmj.com/site/about/guidelines.xhtml

They considered that perineal pain from episiotomy is usually tolerable and does not last long, unless there is something wrong such as infection or stitches that could not be absorbed, which is very rare in their views. The doctors did not mention the effects with breastfeeding. On the other hand, the community health care providers and midwives who confirmed the difficulties of breastfeeding. Some commented that some women had to breastfeed in a painful sitting position because they didn't know how to feed baby in any other way; and that the sitting posture was the proper way for the baby to suck mother's nipples. Postpartum constipation and pain of defecation were all recognized as problems by the health professionals.

"If the wound gets infected because of improperly sterilization during the procedure, it would be very troublesome. The healing will take one to three weeks. In this kind of case, women with episiotomy would be more tortured than those with C-section." (Obstetrician, 28 years of relevant work experience, district MCH hospital.)

"Episiotomy does have impacts on daily activities, such as breastfeeding. Some women are unwilling to breastfeed while lying down, or they just don't know how to breastfeed while lying down. Sometimes, people would feel anxious because of the pain. The milk secretion could also be affected by the pain." (Midwife, 20 years of relevant work experience , secondary general hospital)

3. Long term anxiety is a consequence for some, described as a "psychological shadow"

Several women used the word "psychological shadow" cast by the long-term effects from

For peer review only - http://bmjopen.bmj.com/site/about/guidelines.xhtml

episiotomy. The Chinese word implies a negative experience of suffering or torment that leads to a dread or worries of the future-a bit like the experience of war or a tumultuous personal event. In this research, the word “psychological shadow”, illustrated the complicated mechanisms how episiotomy affected women in a long term. This word contained at least two mechanisms: the fear caused by terrible experience made women avoid the relative things, the other is the miserable experience impaired women’s confidence of similar issue and made them fail to do it. “Psychological shadow” would continue through postpartum sexual life and next childbirth in some women.

3.1 Undesirable and affected sexual life

The impressive pain from perineal wound brought women a fear or worry of postpartum sexual life. A woman even asked her husband to await till one year after childbirth, because she suffered severe pain of episiotomy for nearly two months and feared sexual life might take her back to the nightmare again.

> *“Because of the terrible perineal pain, I asked my husband to resume sexual life a year later. I didn’t dare to do it, because I worried the wound would pain again.” (#16, 32 years old, primipara, two years after childbirth)*

Painful experience after episiotomy also brought women a negative psychological suggestion: there is a cut in vulva and it might hurt again and somehow “changed” the sexual life in the future. Under this negative psychological suggestion, what they thought might lead to what they felt. One woman said her pain with sex might have arisen from

For peer review only - http://bmjopen.bmj.com/site/about/guidelines.xhtml

her anxiety-the psychological shadow, instead of real physical pain. Some responses around resumption of sex and the “psychological shadow” included beliefs that their vagina was damaged and loose and may not ever recover. For these women, they were unwilling to have sexual life and described being permanently “changed” that there had been damage done to their vagina.

“Psychologically, I feel that the vagina cannot recover to original state... you feel the vagina is looser than before. And your spouse also has some psychological barriers to postpartum sexual life. I feel that many mothers who undergo episiotomy will have the shadows of sexual life more or less. The psychological shadow might disappear over time, but I don't know yet.”(#1, 35 years old, multipara, six months after childbirth)

3.2 Less confidence in subsequent vaginal deliveries

The “psychological shadow” also impacted on how women viewed a possible subsequent pregnancy. Women showed less confidence in subsequent vaginal deliveries and expressed their doubts through these questions: whether the episiotomy wound would hinder the process of next vaginal delivery; whether the wound would split again in the next vaginal delivery; or whether they would be subject to another episiotomy. In some cases, “psychological shadow” from episiotomy influenced women’s willing to have another child and brought obvious anxiety during further pregnancy: at least one woman claimed clearly that next time she would ask for a cesarean section to avoid episiotomy. One woman said, “if I had a vaginal birth again, and an episiotomy again. I cannot

For peer review only

imagine what will happen, my vagina would be totally ‘useless’ for sexual life.” The interviews indicated a high degree of anxiety about the long-term physical consequences and reflect how this then itself causes further anxiety. Another multiparous woman also said that she was deeply troubled by the fear of “undergoing episiotomy again” during pregnancy.

" I don’t dare to deliver my second child through normal birth (vaginal delivery). The experience of recovering from the episiotomy was indeed miserable. It really scared me. Maybe not having a second child is better... or maybe I would choose C-section even though it has some negative effects... if I had a vaginal birth again, and an episiotomy again. I cannot imagine what will happen, my vagina would be totally ‘useless’ for sexual life." (#13, 39 years old, primipara, two years after childbirth)

“The doctor directly did the episiotomy at my first childbirth. So I gained some childbirth experience and I was always afraid that I would suffer episiotomy again during this childbirth. There was a psychological shadow when I thought of the childbirth... I was worried about these problems such as deliver again, episiotomy again, miserable recovery of episiotomy. Finally, I still got episiotomy again!” (#29, 30 years old, multipara, six months after childbirth)

The “psychological shadow” is not just about psychological issues since women indeed reported some physical problems. They mentioned the uneven or rough skin of perineal wound and painful intercourse, which affected the enjoyment of sexual life. Several multiparous women who experienced episiotomy twice reported that they need longer

For peer review only - http://bmjopen.bmj.com/site/about/guidelines.xhtml

time to recover from the repeated episiotomy. The health providers were also aware of physical abnormalities following episiotomy and psychological concerns about sexual life. For further pregnancy and childbirth, health providers conceded that women who have a miserable experience of episiotomy can cause women's fear and anxiety over the next delivery but they expressed different reflections on women's concern: most dismissed concerns about subsequent deliveries, one midwife, however, thought the hard scar left from last episiotomy is easy to tear again and slower to heal, if episiotomy isn't done in advance.

"What are the impacts of episiotomy on further childbirth? It is true that it may cast a psychological shadow on those women. If the episiotomy wound from first childbirth is infected or she has a severe tear, she won't dare to have another child, or she might choose C-section."(Obstetrician, 28 years of relevant work experience, district MCH hospital)

"It doesn't matter much because interval between births is generally long. It takes at least one year, right? The skin would recover within a year." (Midwife, 25 years of relevant work experience, district MCH hospital)

"Some people are scar physique (a kind of people who easily have enormous scar). This kind of scar is hard and protuberant so that we fear the wound would tear again during the second childbirth. What's worse, If the scar tears and is sewed up again, it can't heal very well." (Midwife, 25 years of work experience, secondary general hospital)

For peer review only

4. Societal norms assume women will not complain

There are specific social norms that pain and suffering is a necessary part of childbirth and a trial in women's lifespan. However, these norms ignore some serious cases so that some women undergo unfair criticisms and people-centered services are insufficient in relevant clinical practice and nursing.

4.1 Pain from childbirth is normal and endurable

There are some societal norms or established opinions about vaginal delivery in society: the endurable pain or other discomforts are regarded as a normal part of childbirth and the puerperium, which is every woman's "fate", as an interviewee said. This pain and discomfort are expected to gradually disappear without treatment. Under these societal norms, women felt the expectations that they should not complain much about the "slight and temporary discomforts" but to be strong and endure the pain or discomfort by themselves. Whilst women accepted this, it appeared that this expectation did not take into account the more substantive pain, discomfort and interference with daily life associated with episiotomy (see our first theme) and this is distressing for women.

> *"I would endure the pain and not mention it. It didn't hurt that much. I could still bear with it... it's normal thing, also the fate of every woman." (#29, 30 years old, multipara, six months after childbirth)*

> *"Whenever I said I felt sore of the perineal wound, they would say, 'why you still feel painful after 4 months?' It sounds like I shouldn't be sore. Every time my husband said*

For peer review only - http://bmjopen.bmj.com/site/about/guidelines.xhtml

these words, I would response to him, 'you should get a cut and experience the healing process." (#28, 21 years old, primipara, six months after childbirth)

4.2 Too many complaints incur criticisms

Too many complaints are not expected and might incur criticisms or gossips. Several women mentioned their family members expected them to endure the “a non-severe discomfort”. Indeed, two of them were frightened that if they complained too much they would be judged as “being low-tolerant” (Jiao qi). This word is a pejorative personality trait, which means a person exaggerate something that is slightly uncomfortable. This word refers to people who have “weak minds” and who are rather cowardly. When women expressed or complained the postpartum suffering too much, their families thought that the women were at risk of this weak character trait of being “low-tolerant”. When these types of judgement happened, the women felt upset and unwilling to speak out, suffering alone. Surprisingly, another interviewed woman even regarded the tolerance of pain as the only choice and even boast of her strong character. Thus, the societal norms make some women “suffer alone” and stop them from seeking help.

"I wondered if all the women would have the perineal pain after the childbirth... they [family members], such as my sister in law said that I was a bit low-tolerant... they all had birth experience but they never heard that a puerpera unable to sit down after childbirth... I didn’t see a doctor because my families said every woman would experience pain after childbirth, and the doctor also said my wound healed well... At that time, I felt it was so hard to be a woman." (#16, 32 years old, primipara, two years

For peer review only - http://bmjopen.bmj.com/site/about/guidelines.xhtml

after childbirth)

"I'm not very low-tolerant... some women are too spoiled to bear any pain and they always groan, which I thought it is meaningless. Nobody could replace your sufferings. It's normal thing, also the fate of every woman." (#29, 30 years old, multipara, six months after childbirth)

4.3 Health services might be influenced by the societal norms

Indeed, these societal norms about tolerating pain also manifest in the way health care was provided. People-centered services were inadequate in the procedure and nursing of episiotomy. Most women thought suturing is more painful than being cut; yet some doctors did not check whether women were effectively anaesthetized during the suturing. One woman complained of pain during suturing but was told to “wait-it will be finished soon”; and another was told to stay still. One woman reported the pain was so severe she did move when being sutured, and then blamed herself for the subsequent healing problems because she had moved. The expected tolerance of pain extended to pain relief: a woman asked for pain relief after childbirth but was refused by the doctors with the reason “the level of pain after vaginal birth can be tolerated”.

“The suturing process was more painful. I cannot keep unmoved because the anesthetic effects tailed off later. And the doctor kept telling me not to move, saying that he couldn’t sew up well if I still move. But it was painful and he was sewing up for a long time because my wound was very big...I couldn't stay still, and I didn't know

For peer review only - http://bmjopen.bmj.com/site/about/guidelines.xhtml

whether the stitches were done properly. I don't know if it related to my unabsorbed suturing knot, maybe it resulted from my own body condition (some immune factor). " (#20, 30 years old, primipara, EP with forceps delivery, two months after delivery)

"I felt painful so much! I thought I really needed some treatments to relive the pain but the doctor thought I could endure this kind of pain... I really can't endure it since my wound is very large. I hadn't fallen asleep for several days after childbirth. The pain was so awful!" (#20, 30 years old, primipara, EP with forceps, two months after childbirth)

DISCUSSION

There are few qualitative studies of episiotomy worldwide, and the ones we have identified do not differentiate between episiotomy alone and perineal trauma (including episiotomy and severe tear). Most qualitative studies focused on episiotomy only were usually conducted in hospital settings and concerned with shorter term consequences of episiotomy.[34-37] By contrast, we identified more information about women's perspectives and personal reflections in the community settings. The description of the "psychological shadow" seems an apt way to describe both the physical and psychological consequences, and how these play out together-for example with dyspareunia, where anxiety may worsen the physical experience.

The limitation of our study included, first, some women were interviewed more than six

For peer review only - http://bmjopen.bmj.com/site/about/guidelines.xhtml

months after childbirth, which might introduce the recall bias for their experiences shortly after episiotomy. Second, we interviewed the women at the different timelines after childbirth instead of performing at the three specified timelines for all participants, so we cannot know the duration of some women's suffering and the shift of their understanding of episiotomy.

When we set out, we anticipated the health care providers would validate women's perceptions, but they seemed unaware of the long-term consequences, and tended to underestimate the degree of pain and restricted function that women reported.

Episiotomy results in extensive physical discomfort for some, and life troubles to solve. In this study, we confirmed women did suffer the perineal pain or discomfort, consistent with qualitative and quantitative studies.[31 38 39] In addition, women in this study complained more about the unabsorbed stitches or split of stitches. Many reviews also indicate there are a part of women need removal or re-suture services due to factors of materials or skills.[40 41] Women in this study also reported episiotomy limited postpartum daily activities including sitting, breastfeeding, defecation, and intercourse.

We have found few studies reporting that episiotomy interferes with breastfeeding. Chou mentioned the perineal pain can interfere the initiation of breastfeeding,[42] and Persico found the exclusive breastfeeding rate of women with episiotomy in first day after delivery was lower than the women with intact perineum.[43] These physical symptoms or morbidity can also cause psychological burden or anxiety. A study in Jordan reported there was an association between post-partum depression and 15 health problems of obstetric,

For peer review only - http://bmjopen.bmj.com/site/about/guidelines.xhtml

gynecologic (that is, episiotomy pain, infection), and general health conditions (including fatigue and headache).[44] These physical problems might have cumulative effects, as a prospective study indicated high burden of breastfeeding problems alone or with co-morbid physical problems was associated with poor maternal mood at 8 weeks, while the high burden of physical health problems was not significantly associated.[45]

The influence on mood also may relate to sexual life, further delivery, and the impact on sex has been reported elsewhere.[46 47] Our findings highlighted that some women with episiotomy feared, or wanted to avoid another pregnancy because of the pain they experienced, or that they would choose C-section in the next childbirth. This is consistent with other qualitative studies about vaginal childbirth,[48 49] and a study from Turkey also indicated fear about impending childbirth can increase the likelihood of requesting a caesarean section.[50]

Episiotomy was administered in this study with women not even knowing it was going to happen. This lack of informed consent appears widespread and is reported in other studies. One study in Brazil mentioned half of interviewed women did not receive any information about the procedure before or during childbirth.[35] Another study reported that the procedure was informed but lack of authorization or was even practiced directly without any explaination.[36] Some women even did not know whether an episiotomy or spontaneous tear was done, and only noticed a greater discomfort during suturing.[34] Women particularly lacked the knowledges about the consequences of episiotomy in our study, one qualitative study about perineal trauma also identified the similar theme “being

For peer review only

For peer review only - http://bmjopen.bmj.com/site/about/guidelines.xhtml

unaware of the episiotomy's consequences".[35]

Women, their families, and even some health professionals in this region also showed little understanding of some of the possible consequences of episiotomy. This opinion is consistent with a systematic mixed studies review about perineal trauma reported the theme "normalization and feeling dismissed", which means women's health problems are regarded as a normal consequence after childbirth and their questions keep unanswered by health professionals.[51] Some studies reported women felt frustrated and abandoned because of the "dismissed by health care providers".[52 53]

The study also raised the interplay between physical injury and pain, the societal expectations that this was normal, and the women's personal anxieties about the anticipated damage to their genitalia and anticipated pain with sex. When a woman with both physical pain and anxiety are not expected to complain, this can make matters worse. These factors and interactions are particularly important in China where the episiotomy rates remain high.

CONCLUSION

Women were inadequately informed about episiotomy, but experienced consequences of the procedure, including pain, interference with daily life. These were compounded by social norms that expect them not to complain and longer-term anxiety about the physical and psychological effects on them as women.

For peer review only - http://bmjopen.bmj.com/site/about/guidelines.xhtml

For peer review only

Acknowledgments The authors thank the valuable contribution of the women and health care providers who participated in this study. The authors are very grateful to Qinjin Huang for her arrangements of study settings. The authors would like to thank Chunyi Gu and Yao Chen for the professional guidance and data analysis. The authors would also like to thank the following health staffs for their assistance of recruitment: Xiaoxing Dai, Jiali Zhang, Hongxia Cui, Linlin Li.

Author Contributions SH, HJ and XQ designed the study and analyzed data. SH and HJ drafted the paper, and XQ revised it. PG helped with analysis, commented on interpretation and helped write the manuscript. SH and HJ contributed equally to this study, who should be regarded as co-first authors. All authors have verified and approved the final version of the abstract for publication.

Competing interests None declared.

Funding This work received support from the Effective Health care Research Consortium, funded by UK aid from the UK government for the benefit of developing countries (Grant: 5242)

Details of ethics approval All participants were informed about the research purpose and contents. Interviews were conducted after written informed consent obtained from each participant. The research obtained the approval from the Institutional Review Board in School of Public Health, Fudan University (ID: 2017-12-0648)

For peer review only - http://bmjopen.bmj.com/site/about/guidelines.xhtml

Provenance and peer review Not commissioned; externally peer reviewed.

Data sharing statement Data are available upon reasonable request.

Open access This is an open access article distributed in accordance with the Creative Commons Attribution Non Commercial (CC BY-NC 4.0) license, which permits others to distribute, remix, adapt, build upon this work non-commercially, and license their derivative works on different terms, provided the original work is properly cited, appropriate credit is given, any changes made indicated, and the use is non-commercial. See: http:// creative commons. org/licenses/by-nc/4.0/.

References:

1. Longo LD. Classic pages in obstetrics and gynecology. A treatise of midwifery. In three parts by Sir Fielding Ould, Dublin, O. Nelson & C. Connor, 1742. *Am J Obstet Gynecol* 1976;124:656.

2. Muhleman MA, Aly I, Walters A, et al. To cut or not to cut, that is the question: A review of the anatomy, the technique, risks, and benefits of an episiotomy. *Clin Anat* 2017;30:362-72.

3. Jiang H, Qian X, Carroli G, et al. Selective versus routine use of episiotomy for vaginal birth. *Cochrane Database Syst Re* 2017;8:2: CD000081.

4. WHO recommendations: intrapartum care for a positive childbirth experience. Geneva: World Health Organization; 2018;150-155.

5. Overview | Intrapartum care for healthy women and babies | Guidance | NICE. Avaulable at: https://www.nice.org.uk/guidance/cg190. (Accessed 13 Jul 2019).

6. ACOG. ACOG Practice Bulletin. Episiotomy. Clinical Management Guidelines for Obstetrician-Gynecologists. Number 71, April 2006. *Obstet Gynecol* 2006;107:957-62.

7. RCOG. The Management of Third- and Fourth-Degree Perineal Tears. Green-top Guideline No. 29.(June. 2015).

8. Blondel B, Alexander S, Bjarnadottir RI, et al. Variations in rates of severe perineal tears and episiotomies in 20 European countries: a study based on routine national data in Euro-Peristat Project. *Acta Obstet Gynecol Scand* 2016;95:746-54.

9. Binfa L, Pantoja L, Ortiz J, et al. Midwifery practice and maternity services: A multisite descriptive study in Latin America and the Caribbean. *Midwifery* 2016;40:218-25.

For peer review only - http://bmjopen.bmj.com/site/about/guidelines.xhtml

10. Kaddoura R, DeJong J, Zurayk H, et al. Episiotomy practice in the Middle East: A Lebanese teaching tertiary care center experience. *Women Birth* 2019;32:223-228.

11. Schantz C, Sim KL, Ly EM , et al. Reasons for routine episiotomy: A mixed-methods study in a large maternity hospital in Phnom Penh, Cambodia. *Reprod Health Matters* 2015;23:68-77.

12. Qian X, Smith H, Zhou L, et al. Evidence-based obstetrics in four hospitals in China: An observational study to explore clinical practice, women's preferences and provider's views. *BMC Pregnancy Childbirth* 2001, 1(1):1.

13. Qian X, Smith H, Liang, J, et al. Evidence-informed obstetric practice during normal birth in China: trends and influences in four hospitals. *BMC Health Serv Res* 2006; 6: 29

14. Wang Q, Zheng S, Ni Y, et al. The effect of labor epidural analgesia on maternal－fetal outcomes: a retrospective cohort study. *Arch Gynecol Obstet* 2018;298:89-96.

15. Tung CW, Cheon WC, Tong WM, et al. Incidence and Risk Factors of Obstetric Anal Sphincter Injuries after Various Modes of Vaginal Deliveries in Chinese Women. *Chin Med J (Engl)* 2015;128:2420-5.

16. Yang X, Zhang HX, Yu HY, et al. The prevalence of fecal incontinence and urinary incontinence in primiparous postpartum Chinese women. *Eur J Obstet Gynecol Reprod Biol* 2010;152:214-7.

17. Zhao Y, Xiao M, Tang F, et al. The effect of water immersion delivery on the strength of pelvic floor muscle and pelvic floor disorders during postpartum period. *Medicine (Baltimore)* 2017;96:8124.

18. Zhu L, Li L, Lang J, et al. Prevalence and risk factors for peri- and postpartum urinary incontinence in primiparous women in China: a prospective longitudinal study. *Int Urogynecol J* 2012;23:563-72.

19. Liang L, Min L, Li N. [Survey on episiotomy among puerperal women in vaginal delivery from 2011 to 2014]. *Maternal and Child Health care of China* 2015;07:1087-9.

20. http://www.stats.gov.cn/tjsj/zxfb/201908/t20190822_1692898.html. (Accessed 22 Nov 2019).

21. Obstetrics Group, Obstetrics and Gynecology Society, Chinese Medical Association. [Clinical guidelines for operative vaginal delivery (2016)]. *Chin J Obstet Gynecol* 2016;51:565-7.

22. O'Brien BC, Harris IB, Beckman TJ, et al. Standards for reporting qualitative research: a synthesis of recommendations. *Acad Med* 2014, 89:1245-51.

23. Yang W. [Status of intrapartum services on evidence-based practice in Pudong Maternal and Child Health Hospital]. Fudan university 2013.

24. Ye Q，Niu L，Zhao L.[Investigation of Puerpera's Pelvic Floor Function and Early Effect of Obstetric Factors on Pelvic Floor Function]. *Journal of Practical Obstetrics and Gynecology* 2016;32:843-6

25. Zhuang W, Li Y, Wu N, et al. [Effects of Midwife Hierarchical Management mode on Improving the Quality Control of the Delivery Room]. *Nurs J Chin PLA* 2013; 30:52-4.

For peer review only - http://bmjopen.bmj.com/site/about/guidelines.xhtml

26. Glaser BAAS. The discovery of grounded theory: Strategies for qualitative research. New York: Aldine Publishing Company 1967

27. Guest G, Bunce A, Johnson L. How Many Interviews Are Enough? *Field Method* 2016;18(1):59-82.

28. Collumbien M, Busza J, Cleland J, et al. Social science methods for research on sexual and reproductive health. Malta: World Health Organization 2012.

29. Tully KP, Stuebe AM, Verbiest SB. The fourth trimester: a critical transition period with unmet maternal health needs. *Am J Obstet Gynecol* 2017;217:37-41.

30. Fahey JO, Shenassa E. Understanding and Meeting the Needs of Women in the Postpartum Period: The Perinatal Maternal Health Promotion Model. *J Midwifery Womens Health* 2013;58:613-21.

31. Dudley L, Kettle C, Waterfield J, et al. Perineal resuturing versus expectant management following vaginal delivery complicated by a dehisced wound (PREVIEW): a nested qualitative study. *BMJ Open* 2017;7:e13008.

32. Burnard P, Gill P, Stewart K, et al. Analysing and presenting qualitative data. *Br Dent J* 2008;204:429-32.

33. Themes and codes. In: Guest, G., MacQueen, K. M., & Namey, E. E. Applied thematic analysis. Thousand Oaks, CA: SAGE Publications 2012:49-78

34. Wey CY, Salim NR, Junior HPDO, et al. The practice of episiotomy: a qualitative study on perceptions of a group of women. *Online Brazilian Journal of Nursing* 2011;10;2. http://www.objnursing.uff.br/index.php/nursing/rt/printerFriendly/3332/html_2

35. Dengo VAR, Dos Santos Silva R, Souza SRRK, et al. Puerperal women's perceptions about episiotomy. *Cogitare Enferm* 2016;21:1-8.

36. Figueiredo G, Barbieri M, Gabrielloni MC, et al. Episiotomy: perceptions from adolescent puerperae. *Invest Educ Enferm* 2015;33:365-73.

37. Previatti JF, de Souza KV. [Episiotomy: focusing women's view]. *Rev Bras Enferm* 2007;60:197-201.

38. Gün İ, Doğan B, Özdamar Ö. Long- and short-term complications of episiotomy. *Turk J Obstet Gynecol* 2016;13:144-8.

39. Manresa M, Pereda A, Bataller E, et al. Incidence of perineal pain and dyspareunia following spontaneous vaginal birth: a systematic review and meta-analysis. *Int Urogynecol J* 2019;30:853-868.

40. Kettle C, Dowswell T, Ismail KM. Continuous and interrupted suturing techniques for repair of episiotomy or second-degree tears. *Cochrane Database Syst Rev* 2012;11: CD000947.

41. Selo-Ojeme DO, Okonkwo CA, Atuanya C, et al. Single-knot versus multiple-knot technique of perineal repair: a randomised controlled trial. *Arch Gynecol Obstet* 2016;294:945-52.

42. Chou D, Abalos E, Gyte GM, et al. Drugs for perineal pain in the early postpartum period

For peer review only

For peer review only - http://bmjopen.bmj.com/site/about/guidelines.xhtml

(Protocol). *Cochrane Database Syst Rev* 2016;3: CD007734.

43. Persico G, Vergani P, Cestaro C, et al. Assessment of postpartum perineal pain after vaginal delivery: prevalence, severity and determinants. A prospective observational study. *Minerva Ginecol* 2013;65:669-78.

44. Safadi RR, Abushaikha LA, Ahmad MM. Demographic, maternal, and infant health correlates of post-partum depression in Jordan. *Nurs Health Sci* 2016;18:306-13.

45. Cooklin AR, Amir LH and Nguyen CD, et al. Physical health, breastfeeding problems and maternal mood in the early postpartum: a prospective cohort study. *Arch Womens Ment Health* 2018;21:365-374.

46. Fodstad K, Staff AC, Laine K. Sexual activity and dyspareunia the first year postpartum in relation to degree of perineal trauma. *Int Urogynecol J* 2016;27:1513-23.

47. Klein MC, Gauthier RJ, Robbins JM, et al. Relationship of episiotomy to perineal trauma and morbidity, sexual dysfunction, and pelvic floor relaxation. *Am J Obstet Gynecol* 1994;171:591-8.

48. Salmon D. A feminist analysis of women's experiences of perineal trauma in the immediate post-delivery period. *Midwifery* 1999;15:247-56.

49. İlhan G, Verit Atmaca FF, Eken M, et al. What is Turkish women's opinion about vaginal delivery? *Turk J Obstet Gynecol* 2015;12:75-8.

50. Serçekuş P and Okumuş H.Fears associated with childbirth among nulliparous women in Turkey. *Midwifery* 2009;25:155-162.

51. Crookall R, Fowler G, Wood C, et al. A systematic mixed studies review of women's experiences of perineal trauma sustained during childbirth. *J Adv Nurs* 2018 May 23. DOI: 10.1111/jan.13724

52. Priddis H, Schmied V, Dahlen H. Women's experiences following severe perineal trauma: a qualitative study. *BMC Womens Health* 2014;14:32.

53. Williams A, Lavender T, Richmond DH, et al. Women's experiences after a third-degree obstetric anal sphincter tear: a qualitative study. *Birth* 2005;32:129-36.

For peer review only

For peer review only - http://bmjopen.bmj.com/site/about/guidelines.xhtml

Reporting checklist for qualitative study.

Based on the SRQR guidelines.

Instructions to authors

Complete this checklist by entering the page numbers from your manuscript where readers will find each of the items listed below.

Your article may not currently address all the items on the checklist. Please modify your text to include the missing information. If you are certain that an item does not apply, please write "n/a" and provide a short explanation.

Upload your completed checklist as an extra file when you submit to a journal.

In your methods section, say that you used the SRQR reporting guidelines, and cite them as:

O'Brien BC, Harris IB, Beckman TJ, Reed DA, Cook DA. Standards for reporting qualitative research: a synthesis of recommendations. Acad Med. 2014;89(9):1245-1251.

		Reporting Item	Page Number
**Title**			
	#1	Concise description of the nature and topic of the study identifying the study as qualitative or indicating the approach (e.g. ethnography, grounded theory) or data collection methods (e.g. interview, focus group) is recommended	1
**Abstract**			
	#2	Summary of the key elements of the study using the abstract format of the intended publication; typically includes background, purpose, methods, results and conclusions	3
**Introduction**			
Problem formulation	#3	Description and significance of the problem / phenomenon studied: review of relevant theory and empirical work; problem statement	5

For peer review only

Purpose or research question	#4	Purpose of the study and specific objectives or questions	6
**Methods**			
Qualitative approach and research paradigm	#5	Qualitative approach (e.g. ethnography, grounded theory, case study, phenomenolgy, narrative research) and guiding theory if appropriate; identifying the research paradigm (e.g. postpositivist, constructivist / interpretivist) is also recommended; rationale. The rationale should briefly discuss the justification for choosing that theory, approach, method or technique rather than other options available; the assumptions and limitations implicit in those choices and how those choices influence study conclusions and transferability. As appropriate the rationale for several items might be discussed together.	6,7
Researcher characteristics and reflexivity	#6	Researchers' characteristics that may influence the research, including personal attributes, qualifications / experience, relationship with participants, assumptions and / or presuppositions; potential or actual interaction between researchers' characteristics and the research questions, approach, methods, results and / or transferability	10,11
Context	#7	Setting / site and salient contextual factors; rationale	7
Sampling strategy	#8	How and why research participants, documents, or events were selected; criteria for deciding when no further sampling was necessary (e.g. sampling saturation); rationale	7,8
Ethical issues pertaining to human subjects	#9	Documentation of approval by an appropriate ethics review board and participant consent, or explanation for lack thereof; other confidentiality and data security issues	8
Data collection methods	#10	Types of data collected; details of data collection procedures including (as appropriate) start and stop dates of data collection and analysis, iterative process, triangulation of sources / methods, and modification of	8,9

For peer review only

		procedures in response to evolving study findings; rationale	
Data collection instruments and technologies	#11	Description of instruments (e.g. interview guides, questionnaires) and devices (e.g. audio recorders) used for data collection; if / how the instruments(s) changed over the course of the study	8,9
Units of study	#12	Number and relevant characteristics of participants, documents, or events included in the study; level of participation (could be reported in results)	7
Data processing	#13	Methods for processing data prior to and during analysis, including transcription, data entry, data management and security, verification of data integrity, data coding, and anonymisation / deidentification of excerpts	10
Data analysis	#14	Process by which inferences, themes, etc. were identified and developed, including the researchers involved in data analysis; usually references a specific paradigm or approach; rationale	10
Techniques to enhance trustworthiness	#15	Techniques to enhance trustworthiness and credibility of data analysis (e.g. member checking, audit trail, triangulation); rationale	10
**Results/findings**			
Syntheses and interpretation	#16	Main findings (e.g. interpretations, inferences, and themes); might include development of a theory or model, or integration with prior research or theory	11-26
Links to empirical data	#17	Evidence (e.g. quotes, field notes, text excerpts, photographs) to substantiate analytic findings	11-26
**Discussion**			
Intergration with prior work, implications, transferability and contribution(s) to the field	#18	Short summary of main findings; explanation of how findings and conclusions connect to, support, elaborate on, or challenge conclusions of earlier scholarship; discussion of scope of application / generalizability; identification of unique contributions(s) to scholarship in a discipline or field	26

For peer review only

Limitations	#19	Trustworthiness and limitations of findings	26,27
**Other**			
Conflicts of interest	#20	Potential sources of influence of perceived influence on study conduct and conclusions; how these were managed	30
Funding	#21	Sources of funding and other support; role of funders in data collection, interpretation and reporting	30

The SRQR checklist is distributed with permission of Wolters Kluwer © 2014 by the Association of American Medical Colleges. This checklist was completed on 31. July 2019 using https://www.goodreports.org/, a tool made by the EQUATOR Network in collaboration with Penelope.ai

For peer review only

Characteristics of health care providers

	Health care providers
**Age (year)**	
Mean±SD	40.8±5.0
Range	29~52
**Occupation**	
Obstetrician	4
Midwife	5
Community health care provider	3
**Obstetric Institution**	
Municipal MCH Hospital	2
Tertiary general hospital	2
District MCH Hospital	3
Secondary general hospital	3
Community health center	2
**Work experience (year)**	
Mean±SD	16.8±8.2
Range	3~28

For peer review only

Themes and illustrative quotes

Themes	Quotes
Women's views of the procedure vary considerably	"I barely knew anything about episiotomy before the delivery."(#6, 28 years old, primipara, six months after childbirth)<br>"The doctors didn't inform me about the procedure(episiotomy). After childbirth, the woman in the same delivery ward asked me 'did you get episiotomy' and I reply 'what's the episiotomy?' I didn't know it before and I finally realized what the anesthesia and suturing meant at that time." (#9, 33 years old, primipara, four days after childbirth)<br>"I used to wonder what episiotomy is, and only came to know exactly what it is after childbirth··· at that time [When I was cut] I know it -- Oh, this is episiotomy!" (#14, 28 years old, primipara, one week after childbirth)<br>"At that time, I thought, 'Oh my god! They will certainly cut my vulva. The vulva would be ugly and [its function would be] affected!' It sounds scary." (#28, 21 years old, primipara, six months after childbirth)<br>"I think it is necessary to do episiotomy when it can accelerate the progress of labor. But if the baby can be delivered smoothly, episiotomy should be avoided. After all, it is still a surgery." (#11, 30 years old, primipara, two months after childbirth)<br>"The hospital takes episiotomy as a routine practice during normal vaginal birth. I think if episiotomy can relieve your suffering, routine episiotomy should be recommended. I felt that my perineum recovered soon after episiotomy. On the other hand, episiotomy won't cause any big problems, as long as you move carefully and clean yourself frequently." (#26, 28 years old, primipara, two weeks after childbirth)<br>"The doctor said that my uterine contractions were too weak, but I didn't feel that way. I just needed some time. I don't like the episiotomy at all. I searched episiotomy on the Internet and found its rate in China is excessively high. Many situations are not necessary. The doctors might be afraid of potential risks. I think if there is nothing wrong with the puerpera, the episiotomy should be avoided as far as possible. Previous generation like my mother didn't use episiotomy but they recovered quite well." (#8, 34 years old, primipara, two months after childbirth)
The pain interferes with daily life for	"I still feel pain of my perineal wound now and I can feel the difference between the two sides of perineum... the right side with the episiotomy lack skin elasticity... " (#1, 35 years old, multipara, EP with forceps, six

For peer review only - http://bmjopen.bmj.com/site/about/guidelines.xhtml

**weeks**	months after childbirth)<br>"The wound hurt in the first few days. Five days after delivery, I started to feel better, but I can still feel the pulling or tugging pain at the incision… it was a bit tight." (#26, 28 years old, primipara, two weeks after childbirth)<br>" When I tried to sit down, it seems like that I was sitting on a knife. The wound seemed to split. It hurt that much." (#16, 32 years old, primipara, two years after childbirth)<br>"The wound split at the six day after birth, then I suffered a lot because it recovered slowly. The pain had continued for half a month and the stiches cannot be absorbed… even now, I am still feeling painful when I am sitting" (#8, 34 years old, two months after childbirth)<br>"I had to move slowly because I felt painful when I moved suddenly." (#12, 40 years old, multipara, two months after childbirth)<br>"The doctor said I must sleep on the side. It was uncomfortable to sleep one-sided for a long time." (#6, 28 years old, primipara, six months after delivery)<br>"At that time (half a month after childbirth), I couldn't sit or squat [because of the horrible pain], and I had to move very slowly." (#8, 34 years old, two months after childbirth)<br>"The healing was not very good [of my perineum]… in the first few days, I was fed by my mother. I couldn't sit [because of pain], and I just lay down there. I ate on the bed in the first month. " (#20, 30 years old, primipara, EP with forceps delivery, two months after childbirth)<br>"I can't sit because of the pain… in the first month after childbirth, I used the breast pump in a standing position." (#20, 30 years old, primipara, EP with forceps delivery, two months after childbirth)<br>"I just felt the wound painful during breastfeeding. Sometimes I felt that my baby couldn't get the nipples, so I just leaned forward a bit, and suddenly, the episiotomy wound began to hurt. After all, there were two cuts in my perineum [The women also got episiotomy at her last childbirth]… and I don't like to lie down [to feed the baby]." (#4, 34 years old, multipara, two months after childbirth)<br>"It was very tiring and painful to sit down… I felt my wound was also swollen, and I had to sit on one-side, lean my body to the side without episiotomy. I sat in this way for the breastfeeding within the whole first month… this made my back hurt and sometimes it was really awful." (#28, 21 years old, primipara, six months after childbirth)<br>"My wound hurt very much in the first week, and I couldn't peep or poop at all because I couldn't sit on the toilet. Every time using the toilet was

For peer review only - http://bmjopen.bmj.com/site/about/guidelines.xhtml

	like a torture to me. I think that most women who have received an episiotomy would probably have the same problem as me." (#1, 35 years old, multipara, EP with forceps, six months after childbirth)<br>"If the wound gets infected because of improperly sterilization during the procedure, it would be very troublesome. The healing will take one to three weeks. In this kind of case, women with episiotomy would be more tortured than those with C-section." (Obstetrician, 28 years of relevant work experience, district MCH hospital.)<br>"Catgut (a kind of stitches) absorption varies from people to people, and some women may be allergic··· have catgut rejection. If the sutures cannot be absorbed, it is always a foreign matter in perineum, which will hurt. " (Midwife, 25 years of relevant work experience, district MCH hospital)<br>"Episiotomy does have impacts on daily activities, such as breastfeeding. Some women are unwilling to breastfeed while lying down, or they just don't know how to breastfeed while lying down. Sometimes, people would feel anxious because of the pain. The milk secretion could also be affected by the pain." (Midwife, 20 years of relevant work experience, secondary general hospital)<br>"Walking and breastfeeding could be affected [by EP]. Episiotomy has a major impact on breastfeeding, because some women have short nipples, and it is difficult for the baby to suck the nipples when in lying position. Thus, it is easier to breastfeed with sitting position (Community health care provider, 17 years of relevant work experience, community health center)
**Long term anxiety is a consequence for some, described as a "psychological shadow"**	"Because of the terrible perineal pain, I asked my husband to resume sexual life a year later. I didn't dare to do it, because I worried the wound would pain again." (#16, 32 years old, primipara, two years after childbirth)<br>"We returned to sexual life quite late, about half a year after delivery. The first three or four times [of sexual life] turned out painful because of the wound and vaginal dryness. When my baby was 10 months old, it wasn't painful anymore." (#23, 29 years old, primipara, 14 months after childbirth)<br>"Psychologically, I feel that the vagina cannot recover to original state···you feel the vagina is looser than before. And your spouse also has some psychological barriers to postpartum sexual life. I feel that many mothers who undergo episiotomy will have the shadows of sexual life more or less. The psychological shadow might disappear over time, but I

For peer review only - http://bmjopen.bmj.com/site/about/guidelines.xhtml

	don't know yet."(#1, 35 years old, multipara, six months after childbirth)<br><br>"The biggest problem for me now is the next delivery. Will I receive an episiotomy again? Will my second vaginal delivery encounter difficulties because of my rough perineum [the wound of episiotomy this time]?" (#3, 32 years old, primipara, EP with forceps, 14 months after childbirth)<br><br>" I don't dare to deliver my second child through normal birth (vaginal delivery). The experience of recovering from the episiotomy was indeed miserable. It really scared me. Maybe not having a second child is better… or maybe I would choose C-section even though it has some negative effects… if I had a vaginal birth again, and an episiotomy again. I cannot imagine what will happen, my vagina would be totally 'useless' for sexual life." (#13, 39 years old, primipara, two years after childbirth)<br><br>"The doctor directly did the episiotomy at my first childbirth. So I gained some childbirth experience and I was always afraid that I would suffer episiotomy again during this childbirth. There was a psychological shadow when I thought of the childbirth… I was worried about these problems such as deliver again, episiotomy again, miserable recovery of episiotomy. Finally, I still got episiotomy again!" (#29, 30 years old, multipara, six months after childbirth)<br><br>"We used the method of withdraw when having sex, because the inside of my perineum was uneven and rough, which hooked the condom and affected intercourse." (#13, 39 years old, primipara, seven years after childbirth)<br><br>"Some women can't have intercourse at all. Once I met a case that the vagina of the women so tight that I can' even put one finger in, due to the scar contracture." (Obstetrician,28 years of relevant work experience, district MCH hospital)<br><br>"What are the impacts of episiotomy on further childbirth? It is true that it may cast a psychological shadow on those women. If the episiotomy wound from first childbirth is infected or she has a severe tear, she won't dare to have another child, or she might choose C-section. Next time she might say: 'I don't want another baby' and 'I want to choose C-section instead of vaginal delivery."(Obstetrician, 28 years of relevant work experience, district MCH hospital)<br><br>"It doesn't matter much because interval between births is generally long. It takes at least one year, right? The skin would recover within a year… it's just that the wound will look ugly but the birth process won't be affected." (Midwife, 25 years of relevant work experience, district MCH hospital)<br><br>"Some people are scar physique (a kind of people who easily have enormous scar). This kind of scar is hard and protuberant so that we fear

	the wound would tear again during the second childbirth. If the scar tears and is sewed up again, it can't heal very well. (Midwife, 25 years of work experience, secondary general hospital)
Societal norms assume women will not complain	"I would endure the pain and not mention it. It didn't hurt that much. I could still bear with it... It's normal thing, also the fate of every woman." (#29, 30 years old, multipara, six months after childbirth)<br><br>"Whenever I said I felt sore of the perineal wound, they would say, 'why you still feel painful after 4 months?' It sounds like I shouldn't be sore. Every time my husband said these words, I would response to him, 'you should get a cut and experience the healing process." (#28, 21 years old, primipara, six months after childbirth)<br><br>"I wondered if all the women would have the perineal pain after the childbirth... they [family members], such as my sister in law said that I was a bit low-tolerant... they all had birth experience but they never heard that a puerpera unable to sit down after childbirth... I didn't see a doctor because my families said every woman would experience pain after childbirth, and the doctor also said my wound healed well... At that time, I felt it was so hard to be a woman." (#16, 32 years old, primipara, two years after childbirth)<br><br>"I'm not very low-tolerant... some women are too spoiled to bear any pain and they always groan, which I thought it is meaningless. Nobody could replace your sufferings. It's normal thing, also the fate of every woman." (#29, 30 years old, multipara, six months after childbirth)<br><br>"The suturing process was more painful. I cannot keep unmoved because the anesthetic effects tailed off later. And the doctor kept telling me not to move, saying that he couldn't sew up well if I still move. But it was painful and he was sewing up for a long time because my wound was very big...I couldn't stay still, and I didn't know whether the stitches were done properly. I don't know if it related to my unabsorbed suturing knot, maybe it resulted from my own body condition (some immune factor). " (#20, 30 years old, primipara, EP with forceps delivery, two months after delivery)<br><br>"It was painful during suturing. It was the sharp tingling when I got the cut but the pain of the childbirth was more painful than this. After local anesthetic, it was still very painful and he sewed a few stitches. But I was immersed in the joy of having a baby at that time so I felt the pain could be tolerated···but I was trembling because of pain and the doctor comforted me 'it would be finished soon, you can bear it, yes?" (#23, 29 years old, primipara, 14 months after delivery)<br><br>"I felt painful so much! I thought I really needed some treatments to relive

For peer review only - http://bmjopen.bmj.com/site/about/guidelines.xhtml

	the pain but the doctor thought I could endure this kind of pain… I really can't endure it since my wound is very large. I hadn't fallen asleep for several days after childbirth. The pain was so awful!" (#20, 30 years old, primipara, EP with forceps, two months after childbirth)

For peer review only

For peer review only - http://bmjopen.bmj.com/site/about/guidelines.xhtml
